# Bacterial Signatures of Paediatric Respiratory Disease: An Individual Participant Data Meta-Analysis

**DOI:** 10.3389/fmicb.2021.711134

**Published:** 2021-12-23

**Authors:** David T. J. Broderick, David W. Waite, Robyn L. Marsh, Carlos A. Camargo, Paul Cardenas, Anne B. Chang, William O. C. Cookson, Leah Cuthbertson, Wenkui Dai, Mark L. Everard, Alain Gervaix, J. Kirk Harris, Kohei Hasegawa, Lucas R. Hoffman, Soo-Jong Hong, Laurence Josset, Matthew S. Kelly, Bong-Soo Kim, Yong Kong, Shuai C. Li, Jonathan M. Mansbach, Asuncion Mejias, George A. O’Toole, Laura Paalanen, Marcos Pérez-Losada, Melinda M. Pettigrew, Maxime Pichon, Octavio Ramilo, Lasse Ruokolainen, Olga Sakwinska, Patrick C. Seed, Christopher J. van der Gast, Brandie D. Wagner, Hana Yi, Edith T. Zemanick, Yuejie Zheng, Naveen Pillarisetti, Michael W. Taylor

**Affiliations:** ^1^School of Biological Sciences, University of Auckland, Auckland, New Zealand; ^2^Child Health Division, Menzies School of Health Research, Charles Darwin University, Darwin, NT, Australia; ^3^Department of Emergency Medicine, Massachusetts General Hospital, Boston, MA, United States; ^4^Department of Epidemiology, Harvard T.H. Chan School of Public Health, Boston, MA, United States; ^5^Harvard Medical School, Boston, MA, United States; ^6^Colegio de Ciencias Biológicas y Ambientales, Instituto de Microbiología, Universidad San Francisco de Quito, Quito, Ecuador; ^7^Department of Respiratory and Sleep Medicine, Queensland Children’s Hospital, Brisbane, QLD, Australia; ^8^Australian Centre for Health Services Innovation, Queensland University of Technology, Brisbane, QLD, Australia; ^9^National Heart and Lung Institute, Imperial College London, London, United Kingdom; ^10^Royal Brompton and Harefield NHS Foundation Trust, London, United Kingdom; ^11^Department of Obstetrics and Gynecology, Peking University Shenzhen Hospital, Shenzhen, China; ^12^School of Medicine, University of Western Australia, Perth, WA, Australia; ^13^Department of Pediatrics, Gynecology and Obstetrics, Faculty of Medicine, University Hospitals of Geneva, Geneva, Switzerland; ^14^Department of Pediatrics, University of Colorado School of Medicine, Aurora, CO, United States; ^15^Seattle Children’s Hospital, Seattle, WA, United States; ^16^Department of Pediatrics and Microbiology, University of Washington, Seattle, WA, United States; ^17^Department of Pediatrics, Childhood Asthma Atopy Center, Humidifier Disinfectant Health Center, Asan Medical Center, University of Ulsan College of Medicine, Seoul, South Korea; ^18^Hospices Civils de Lyon, Lyon, France; ^19^Division of Pediatric Infectious Diseases, Duke University, Durham, NC, United States; ^20^Department of Life Science, Multidisciplinary Genome Institute, Hallym University, Chuncheon, South Korea; ^21^Department of Biostatistics, Yale School of Public Health, Yale University, New Haven, CT, United States; ^22^Department of Computer Science, City University of Hong Kong, Kowloon, Hong Kong SAR, China; ^23^Department of Biomedical Engineering, City University of Hong Kong, Kowloon, Hong Kong SAR, China; ^24^Department of Pediatrics, Boston Children’s Hospital, Boston, MA, United States; ^25^Division of Pediatric Infectious Diseases, Department of Pediatrics, Center for Vaccines and Immunity, Abigail Wexner Research Institute at Nationwide Children’s Hospital, The Ohio State University College of Medicine, Columbus, OH, United States; ^26^Department of Microbiology and Immunology, Geisel School of Medicine at Dartmouth, Hanover, NH, United States; ^27^Finnish Institute for Health and Welfare (THL), Helsinki, Finland; ^28^Department of Biostatistics and Bioinformatics, Computational Biology Institute, Milken Institute School of Public Health, George Washington University, Washington, DC, United States; ^29^CIBIO-InBIO, Centro de Investigação em Biodiversidade e Recursos Genéticos, Universidade do Porto, Campus Agrário de Vairão, Vairão, Portugal; ^30^Department of Epidemiology of Microbial Diseases, Yale School of Public Health, New Haven, CT, United States; ^31^CHU Poitiers, Infectious Agents Department, Poitiers, France; ^32^University of Poitiers, INSERM U1070, Poitiers, France; ^33^Department of Biosciences, University of Helsinki, Helsinki, Finland; ^34^Nestlé Research, Lausanne, Switzerland; ^35^Department of Pediatrics, Feinberg School of Medicine, Northwestern University, Chicago, IL, United States; ^36^Department of Life Sciences, Manchester Metropolitan University, Manchester, United Kingdom; ^37^Department of Biostatistics and Informatics, Colorado School of Public Health, University of Colorado, Aurora, Aurora, CO, United States; ^38^School of Biosystem and Biomedical Science, Korea University, Seoul, South Korea; ^39^Shenzhen Children’s Hospital, Shenzhen, China; ^40^Starship Children’s Hospital, Auckland, New Zealand

**Keywords:** microbiota (16S), respiratory tract, respiratory infection, paediatrics, meta-analysis, individual participant data (IPD) meta-analysis

## Abstract

**Introduction:** The airway microbiota has been linked to specific paediatric respiratory diseases, but studies are often small. It remains unclear whether particular bacteria are associated with a given disease, or if a more general, non-specific microbiota association with disease exists, as suggested for the gut. We investigated overarching patterns of bacterial association with acute and chronic paediatric respiratory disease in an individual participant data (IPD) meta-analysis of 16S rRNA gene sequences from published respiratory microbiota studies.

**Methods:** We obtained raw microbiota data from public repositories or *via* communication with corresponding authors. Cross-sectional analyses of the paediatric (<18 years) microbiota in acute and chronic respiratory conditions, with >10 case subjects were included. Sequence data were processed using a uniform bioinformatics pipeline, removing a potentially substantial source of variation. Microbiota differences across diagnoses were assessed using alpha- and beta-diversity approaches, machine learning, and biomarker analyses.

**Results:** We ultimately included 20 studies containing individual data from 2624 children. Disease was associated with lower bacterial diversity in nasal and lower airway samples and higher relative abundances of specific nasal taxa including *Streptococcus* and *Haemophilus*. Machine learning success in assigning samples to diagnostic groupings varied with anatomical site, with positive predictive value and sensitivity ranging from 43 to 100 and 8 to 99%, respectively.

**Conclusion:** IPD meta-analysis of the respiratory microbiota across multiple diseases allowed identification of a non-specific disease association which cannot be recognised by studying a single disease. Whilst imperfect, machine learning offers promise as a potential additional tool to aid clinical diagnosis.

## Introduction

The human respiratory tract has long been of interest to clinicians and microbiologists, with a traditional focus on single, putatively pathogenic microorganisms (Huang and Lynch, 2011). More recent research, enabled by advances in DNA sequencing, has revealed the existence of complex bacterial communities (microbiota) throughout the airways of even healthy individuals (Charlson et al., 2011; Man et al., 2017; Cox et al., 2019). While a protective function of the respiratory microbiota has been proposed (Man et al., 2017), it is increasingly apparent that a microbiota imbalance, or dysbiosis, is frequently associated with disease. Understanding how, or even if, patterns within the microbiota correspond to different respiratory diagnoses is a key challenge.

Respiratory disease in children is a known risk factor for chronic disease in adulthood (Grimwood and Chang, 2015; Bui et al., 2018; Zhang, 2020). As such, any reduction in childhood disease burden could also improve the outlook for adult respiratory health. In particular, respiratory infections in children are associated with future impaired lung function as adults (Zhang, 2020). While connections between paediatric and adult respiratory disease remain enigmatic, it is likely that the airway microbiota plays a role. Indeed, atypical development of the infant upper airway microbiota has been linked to unfavourable respiratory outcomes in older children (Biesbroek et al., 2014; Teo et al., 2015), a pattern which may continue into adulthood. These findings, together with the role of microbes in early life immune education and evidence for a shared core microbiota across different respiratory diseases in children but not adults (van der Gast et al., 2014), suggest a potential therapeutic window aiming at a beneficial microbiota (e.g., *via* probiotics or targetted antibiotics). Although the microbiota of the upper airway may not contribute directly to pathogenesis of lower airway disease, it nonetheless demands consideration both as a reservoir for lower airway microorganisms as well as being a more clinically accessible site.

Studying the respiratory microbiota in children brings challenges including logistical and ethical considerations with sampling the paediatric lower airway and inherent difficulties with low-microbial-biomass samples (Marsh et al., 2018). Consequently, understanding of the paediatric respiratory microbiota is not as developed as for other organs with more accessible specimens, such as the bowel. However, with an ever-increasing body of studies, and a field that is already starting to look beyond the typical sequencing of amplified bacterial 16S rRNA genes, it is both feasible and timely to examine what these data as a whole reveal about the microbiota and paediatric respiratory illness. Indeed, rigorous examination of existing data should serve to guide future application of approaches based on microbial function, such as metagenomics and metatranscriptomics (Ritchie and Singanayagam, 2020).

Variation within the human microbiota is considerable, with differences among individuals sometimes swamping signals from clinical factors such as disease (Biswas et al., 2015). Further challenges arise due to a lack of standardisation among studies, such as anatomical site sampled, collection technique, DNA extraction method, 16S rRNA gene region sequenced, and bioinformatics approach. This signals the need for large sample numbers which are not always feasible in paediatric respiratory studies. By combining individual participant data (IPD) from multiple studies, IPD meta-analyses help identify biologically and/or clinically relevant patterns that may not otherwise be detected in small, unstratified studies. Standardised bioinformatics pipelines for re-processing microbiota data from multiple studies have been successfully applied to the human gut (Duvallet et al., 2017), sinus (Wagner Mackenzie et al., 2017), and cystic fibrosis (CF) lung microbiotas (Li et al., 2016).

The value of contextualising results across multiple diseases was eloquently demonstrated by Duvallet et al. (2017) in a recent IPD meta-analysis of the gut microbiota. As this approach has never been done for respiratory microbiota across a range of acute and chronic childhood diseases, we collated and re-analysed published 16S rRNA gene-based microbiota data from 20 studies encompassing such diagnoses. We aimed to test several hypotheses, namely that: (a) the airway microbiota differs between health and disease; (b) there is a consistent microbiota signature in children with lower airway disease, irrespective of specific diagnosis; and (c) airway microbiota profiles can be used to detect lower airway disease.

## Materials and Methods

### Study Selection Strategy

To identify publications with 16S rRNA gene sequence data from cross-sectional analyses containing paediatric respiratory samples, Scopus and PubMed databases were searched on January 2, 2018 using 25 search terms (Supplementary File A). To be included in the analysis studies could only have a single timepoint per individual within that publication and investigate one of the following illnesses: bronchiolitis, bronchiectasis, CF, asthma, wheeze, acute respiratory infections, chronic suppurative lung disease, and protracted or persistent bacterial bronchitis. Studies which focused on lung transplants, individuals undergoing mechanical ventilation, contained 10 or fewer disease samples, or where age, sex, or diagnosis were unclear, were excluded. We also contributed new, at the time unpublished, data of our own (Pillarisetti et al., 2019). Overall, we obtained data from 4884 samples across 21 studies. Separate ethics approvals had been obtained for each original study and the University of Auckland Human Participants Ethics Committee deemed that re-use of these published data did not require additional approval.

### Sequence Data Processing

We used a uniform bioinformatics pipeline to analyze all of the included studies (code is supplied in Supplementary Material). The analysis pipeline utilised USEARCH (v.11.0667) (Edgar, 2010) for quality filtering and, where applicable, merging of paired-end reads, after which reference gene alignment and chimaera removal (chimera.uchime) (Edgar et al., 2011) were performed using mothur (v1.38.1) (Schloss et al., 2009). Taxonomic classification (classify.otu) was performed in mothur using the SILVA SSU database (v132) as reference (Quast et al., 2012). Sequences assigned to non-bacterial lineages or that could not be identified to genus level were removed. Reprocessing of the data led to removal of 557 samples not containing any reads which could be identified at genus level through our uniform pipeline. The wide range of sequenced 16S rRNA regions (Supplementary File A and Supplementary Figure E1) necessitated a genus-level phylotype approach (Waite and Taylor, 2014; Callahan et al., 2017). Following data reprocessing, our uniformly applied bioinformatics approach was able to successfully recapitulate most testable claims identified in the original papers, across multiple normalisation approaches and rarefaction depths (Supplementary File C). Removal of samples with <1000 sequences (348 total) resulted in removal of a single study (Langevin et al., 2017), as retention of only 8% of their samples meant this study could not be fairly represented. Hence, only 20 studies were ultimately included. Following normalisation, pseudoreplication bias (i.e., over-representation of a particular bacterial community) was avoided by splitting data into four broad anatomical sites with distinct physicochemical features [nasal, oral, sputum, lower airways (bronchoalveolar lavage or bronchial brushings)] (Marsh et al., 2016; Man et al., 2017; Ronchetti et al., 2018), and avoiding cross-site comparisons. Ultimately, 2789 samples (from 2624 individuals) were retained.

### Defining Diagnostic Groupings

Given variations in how clinical diagnoses are reported across studies, we grouped reported diagnoses into separate groups on two levels. The first, broad level grouped diagnoses into (a) controls, comprising individuals deemed healthy or suitable to use as disease controls in the original study; and (b) disease, comprising individuals with diagnosed respiratory disease. For the second, more nuanced level we defined seven mutually exclusive diagnostic groupings: acute infections, asthma, CF, disease control, healthy, suppurative lung diseases (e.g., bronchiectasis, protracted bacterial bronchitis), and wheezing illness.

### Data Analysis

Statistical analyses were performed in R (v3.6.2) (R Core Team, 2019), using vegan (Jari et al., 2015) for diversity calculations, and ggplot2 (Wickham, 2016) for generating plots. Genus-level phylotypes differing significantly between diagnostic groupings were identified using linear discriminant analysis effect size (LEfSe) (Segata et al., 2011). We identified the core microbiota for specific anatomical sites and diagnostic groupings by applying a prevalence threshold of ≥75% in the relevant samples, and an abundance filter whereby a genus must represent ≥10% in at least one sample.

### Sensitivity Analyses

In addition to anatomical site and clinical condition, we attempted to account for other factors affecting the respiratory microbiota. In sensitivity analyses we examined effect of participant age, data normalisation approach and influence of two specific studies on our overall findings. While results shown here are based on rarefaction to 1000 sequence reads/sample, we also considered a size factor-based normalisation approach (GMPR) (Chen et al., 2018). This allowed for an accounting of the flaws in each method, namely data compositionality in rarefaction and bias caused by differing sequencing depths in GMPR. The data from one study (Wang et al., 2016) formed a distinctly separate cluster in ordination (principle coordinates analysis, multidimensional scaling) analyses (Supplementary Figure E6). The second study (Luna et al., 2018) represented such a large proportion of the overall dataset that we had concerns our findings could simply reflect that study’s original findings. To test for any such effects, we: (a) eliminated all samples from a particular category (e.g., a specific age group/study) then repeated the analyses; and (b) retained only samples pertaining to the single category in question and re-analysed these independently. Both approaches aimed to determine whether our initial overall findings could be recapitulated.

### Machine Learning

To determine whether diagnostic groups could be identified based only on microbiota composition, machine learning trials were performed on rarefied data in python (Pedregosa et al., 2011). For this, 60% of samples from a given anatomical site were selected at random to use as training data, with the remaining 40% used for validation. Of five different initial approaches [random forest; neural network; support vector machine (SVM): linear; SVM: radial bias function; SVM: polynomial], random forest was most successful across the majority of trials. Success in this context was determined by the accuracy value (the fraction of correct calls over all calls) (Sokolova et al., 2006). The random forest approach was then applied independently to both the rarefied and GMPR-normalised datasets using the training and validation strategy outlined above. In addition, for both normalisation methods we generated a sample dataset in which the contribution of different diagnostic groupings was set as equivalent to account for differences in sample numbers. These final machine learning approaches were assessed for both their positive predictive value (defined as the fraction of calls of a diagnostic grouping which are correct) and sensitivity (defined as the fraction of samples within a diagnostic grouping which are correctly identified) for all specific diagnostic groupings.

For more details see Supplementary File A.

## Results

**FIGURE 1:**
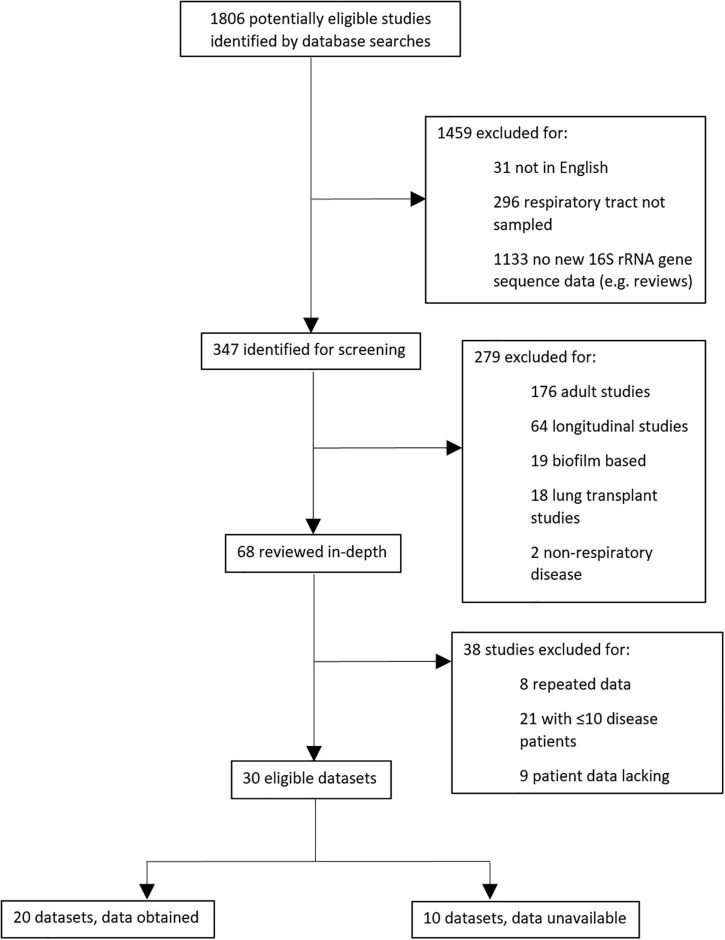
Schematic showing protocol for selecting meta-analysis studies.

In total, 1806 potentially eligible studies were identified by literature database searches, of which 347 required screening and evaluation against specific criteria (Supplementary File A). Thirty eligible datasets were ultimately interrogated, and we were able to access 16S rRNA gene sequences and essential metadata for 20 of these, in addition to our own new data (Pillarisetti et al., 2019; Figure 1). After removal of one study (Langevin et al., 2017) due to low sample retention, ultimately 20 datasets were included.

Bioinformatic processing using a uniform analysis pipeline resulted in a final dataset comprising 2789 samples, from 2624 individuals. Following sequence data reprocessing, the assembled dataset encompassed 20 unique studies (Table 1) spanning chronic and acute diagnoses, multiple anatomical sites, and 11 countries. Technical, clinical, and demographic heterogeneity were considerable (Table 2). While use of a uniform bioinformatics pipeline is expected to reduce some underlying data variation, other technical factors such as sequencing technology used, 16S rRNA gene region sequenced, and DNA extraction method could not be readily controlled. This was evident in that the variables “Study,” “Extraction method,” “16S rRNA gene region,” and “sequencing platform” accounted for 12.4, 6.75, 4.76, and 0.438% of underlying data variation, respectively, according to PERMANOVA analysis (Table 3). The most substantial of these technical factors was DNA extraction method, as accounting for this factor could reduce the explanatory power of “Study” to less than 1% (Table 3). Clinical and demographic heterogeneity reflected the broad range of diagnoses included, ages spanning infancy to near-adulthood (<18 years), and diverse strategies regarding anatomical site sampled and sample collection technique. For many clinically relevant factors, e.g., antibiotics and other medication usage, data were incomplete or inconsistently reported.

**TABLE 1:** Summary of studies which contributed to the final dataset; for more detailed summaries see Supplementary File A (Supplementary Tables E2–E5).

Studys	Age (years)	Sample site(s)	Disease	N disease	Control individuals	N control
Cardenas et al., 2012	0.5–1.083	OP	Early onset wheeze	21	Healthy	23
Cuthbertson et al., 2017	0.8–15.4	Bronchial brushings	PBB	23	Healthy	19
de Steenhuijsen Piters et al., 2016	0.025–1.83	NP	RSV infections	105	Healthy	26
Hampton et al., 2014	9.86–17.58	IS	Cystic fibrosis	13		
Kelly et al., 2017	0.083–1.99	NP	Pneumonia URI symptoms	374 82	Control	90
Kim et al., 2017	6–14	NP	Asthma Asthma remission	26 17	Healthy	21
Lu et al., 2017	0.1–12.7	NP, OP	Pneumonia	120	Healthy	113
Luna et al., 2018	0.027–1	NP	Bronchiolitis	814		
Marsh et al., 2016 *	0.4–10.1	BAL, NP, OP	Bronchiectasis CSLD PBB	46 6 21	Disease control	9
Perez-Losada et al., 2016	6–17	NP	Asthma	29		
Pettigrew et al., 2016	0.50–17.25	IS	Pneumonia	310		
Pillarisetti et al., 2019 *	0.9–16	Anterior nares, BAL	Bronchiectasis	54	Healthy	26
Ruokolainen et al., 2017	14–17	Anterior nares	Asthma	9	Healthy	118
Sakwinska et al., 2014	0.2–5.0	NP	Pneumonia	14	Healthy	2
van der Gast et al., 2014	0.63–16.85	BAL, sputum	Bronchiectasis Cystic fibrosis PBB	12 18 9		
Wang et al., 2016	0.3–9	BAL	Pneumonia	22	Tracheomalacia	12
Williamson et al., 2017	1.5–1.7	ES, OP	CFTR-related Cystic fibrosis	1 68		
Yi et al., 2014	0–13	OP	Acute infection	25		
Zemanick et al., 2015	8.49–17.89	ES, IS, OP, saliva	Cystic fibrosis	30		
Zemanick et al., 2017	0.166–17.0	BAL	Cystic fibrosis	50	Disease control	11

*Denotes inclusion of multiple samples for some individuals. Diagnoses are as reported in the original papers. BAL, bronchoalveolar lavage; CFTR, cystic fibrosis transmembrane conductance regulator; CSLD, chronic suppurative lung disease; ES, expectorated sputum; IS, induced sputum; NP, nasopharyngeal; OP, oropharyngeal; PBB, protracted bacterial bronchitis; RSV, respiratory syncytial virus; URI, upper respiratory infection.

**TABLE 2:** Summary of technical factors associated with each of the included studies.

Studys	Sampling site(s)	Sampling method	% eligible samples retained	DNA extraction method	16S rRNA gene region	Sequencing technology
Cardenas et al., 2012	OP	Swab	91.7	QIAamp	V3–V5	454
Cuthbertson et al., 2017	LA	Brushings	66.7	MPBio FastDNA Spin Kit for Soil	V4	MiSeq
de Steenhuijsen Piters et al., 2016	NP	Swab	99.2	NucliSENS	V5–V7	454
Hampton et al., 2014	SP	Induced	100	Gentra PureGene Yeast/Bact. Kit	V4–V6	454
Kelly et al., 2017 *	NP	Swab	99.6	In-house protocol	V3	MiSeq
Kim et al., 2017	NP	Swab	69.6	PowerMag RNA/DNA Isolation Kit	V1–V3	454
Langevin et al., 2017 **	NP	Unknown	8.3	NucliSENS	V1–V3	MiSeq
Lu et al., 2017	NP	Swab	98.3	PowerSoil	V3–V4	MiSeq
	OP	Swab	97.5	PowerSoil	V3–V4	MiSeq
Luna et al., 2018 ***	NP	Aspirate	99.9	PowerSoil	V4	MiSeq
Marsh et al., 2016	NP	Swab	7.8	QIAamp	V1–V3	454
	OP	Swab	71.8	QIAamp	V1–V3	454
	LA	BAL	26.3	QIAamp	V1–V3	454
Perez-Losada et al., 2016	NP	Aspirate	96.7	QIAamp	V4	MiSeq
Pettigrew et al., 2016 ****	SP	Induced	100	NucliSENS	V4	MiSeq
Pillarisetti et al., 2019	AN	Swab	97.3	Qiagen AllPrep	V3–V4	MiSeq
	LA	BAL	61.4	Qiagen AllPrep	V3–V4	MiSeq
Ruokolainen et al., 2017	AN	Swab	70.2	MPBio FastDNA Spin Kit for Soil	V1–V3	454
Sakwinska et al., 2014 *****	NP	Swab	32.7	In-house protocol	V4	454
van der Gast et al., 2014	LA	BAL	61.9	In-house protocol	V1–V3	454
	SP	Unknown	74.3	In-house protocol	V1–V3	454
Wang et al., 2016	LA	BAL	100	E.Z.N.A Soil DNA Kit	V3–V4	MiSeq
Williamson et al., 2017	OP	Swab	48.8	Qiagen EZ1	V1–V2	MiSeq
	SP	Expectorated	35.9	Qiagen EZ1	V1–V2	MiSeq
Yi et al., 2014	OP	Swab	37	Qiagen AllPrep	V1–V3	454
	NP	Aspirate	47	Qiagen AllPrep	V1–V3	454
Zemanick et al., 2015	SA	Saliva	63.6	Qiagen EZ1	V1–V2	454
	SP	Induced	58.3	Qiagen EZ1	V1–V2	454
	SP	Expectorated	66.7	Qiagen EZ1	V1–V2	454
	OP	Swab	61.5	Qiagen EZ1	V1–V2	454
Zemanick et al., 2017	LA	BAL	66.3	Qiagen EZ1	V1–V2	MiSeq

*AN, anterior nares; LA, lower airways; NP, nasopharynx; OP, oropharynx; SA, saliva; SP, sputum.*

*This study provided more data than were contained within the original paper.

**This study was removed from the analysis due to low sample retention following our processing.

***This study contained both anterior nare and nasopharyngeal samples from the same individual; to avoid pseudoreplication only nasopharyngeal samples were used in the main analysis.

****This study contained both sputum and swab samples, however, only sputum samples were used.

*****This study sequenced two different 16S rRNA gene regions for each sample, however, we selected only the region with the highest average sequencing depth.

**TABLE 3:** PERMANOVA analysis of technical variables amongst microbiota studies included in this meta-analysis.

Variable combination	Variable	% variation explained	*P*-value
Study alone	Study	12.4	0.001
Extraction method	Extraction method	6.75	0.001
16S rRNA gene region	Gene region	4.76	0.001
Sequencing platform	Sequencing platform	0.438	0.001
Extraction method + study	Extraction method	6.75	0.001
	Study	5.62	0.001
Gene region + study	Gene region	4.76	0.001
	Study	7.61	0.001
Sequencing platform + study	Sequencing platform	0.438	0.001
	Study	11.9	0.001
Extraction method + gene region + study	Extraction method	6.75	0.001
	Gene region	4.74	0.001
	Study	0.878	0.001
Extraction method + sequencing platform + study	Extraction method	6.75	0.001
	Sequencing platform	0.416	0.001
	Study	5.2	0.001
Gene region + extraction method + study	Gene region	4.76	0.001
	Extraction method	6.74	0.001
	Study	0.877	0.001
Gene region + sequencing platform + study	Gene region	4.76	0.001
	Sequencing platform	0.0006	0.072
	Study	7.56	0.001
Sequencing platform + extraction method + study	Sequencing platform	0.438	0.001
	Extraction method	6.73	0.001
	Study	5.2	0.001
Sequencing platform + gene region + study	Sequencing platform	0.438	0.001
	Gene region	4.38	0.001
	Study	7.55	0.001

*Percentage of microbiota variation explained was determined using the R^2^ score.*

### Bacterial Diversity in Health and Disease

**FIGURE 2:**
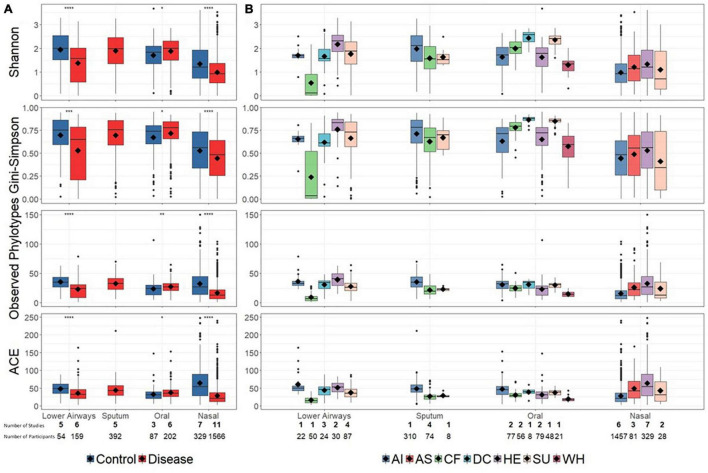
Bacterial alpha-diversity at broad disease **(A)** and specific diagnostic grouping levels **(B)**. Significant differences in **(A)**, as assessed by a Bonferroni corrected *t*-test, are denoted by asterisks (*p* < 0.05). For clarity, statistical significance for **(B)** is presented in Supplementary Tables E6–9. Diamonds on each box represent the mean value. Alpha-diversity across different anatomical sites (lower airways, sputum, oral, and nasal) was not explicitly compared. Below the plot the number of studies (bold) and number of contributing samples is reported. AI, acute infections; AS, asthma; CF, cystic fibrosis; DC, disease control; HE, healthy; SU, suppurative; WH, wheezing illness.

Reduced bacterial diversity, reflecting a lower number and/or uneven distribution of bacterial taxa, is sometimes considered a marker of human disease. We therefore calculated bacterial alpha-diversity on aggregated disease vs. controls data, as well as at a more granular level in which disease and control diagnoses were separated into a total of seven groups (Figure 2). For the aggregated data, alpha-diversity (described by multiple metrics) was significantly higher in controls than disease for nasal and lower airway sites, with the opposite trend for oral samples (Figure 2A). Bacterial richness (observed phylotypes, ACE) differed little between different anatomical sites, though nasal samples did have lower evenness (Shannon, Gini–Simpson), implying dominance by specific taxa. At diagnostic group level (Figure 2B), findings varied depending on anatomical site and whether richness or evenness were considered. One notable finding was that of decreased bacterial diversity in the lower airways of CF patients, although as these data were derived from a single study – and reflect varied pulmonary statuses including both clinical stability and exacerbations at the time of sampling (Zemanick et al., 2017) – one must be circumspect if attempting to infer a wider trend.

### Microbiota Taxonomic Differences Between Health and Disease

**FIGURE 3:**
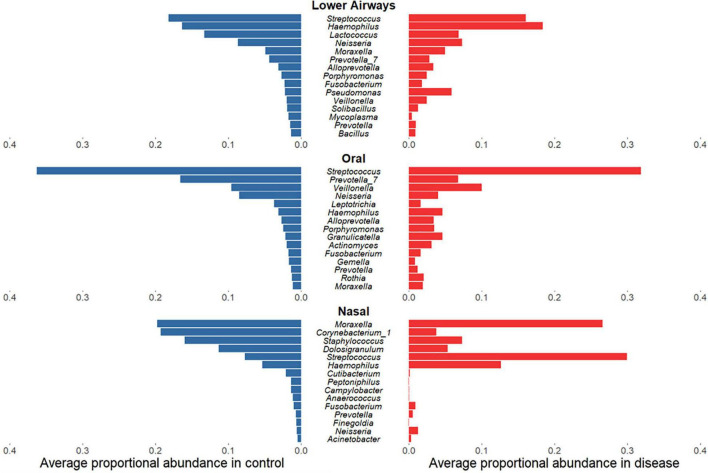
Rank-abundance plots showing the 15 most abundant bacterial phylotypes in control samples (blue) and their respective proportional relative abundance in disease samples (red), for lower airway, oral, and nasal samples. Taxa are ranked based on their relative sequence abundance in controls.

Having established that bacterial diversity differs between respiratory diseases and controls, we sought to determine which bacterial taxa drive these differences. While for oral and lower airway sites the most abundant genera in samples from diseased individuals largely mirrored those from controls (Figure 3), there was far less concordance within the nasal data: disease was associated with clear decreases in relative sequence abundances of *Corynebacterium_1*, *Staphylococcus*, and *Dolosigranulum* but substantial increases in *Streptococcus* and, to a lesser extent, *Haemophilus*. Biomarker analysis of nasal samples *via* LEfSe largely supported these findings, with *Haemophilus* and *Streptococcus* identified as potential markers of disease, while *Corynebacterium_1*, *Staphylococcus*, and *Dolosigranulum* were associated with controls (Supplementary File B and Supplementary Tables E10–16). Observed minor changes in rank-abundance for oral samples were also supported by LEfSe, with *Veillonella* identified as a marker for disease and *Prevotella_7* a marker for controls. In addition to the abundant taxa, rare taxa – phylotypes present in ≤10% of samples for a given anatomical site which never comprise ≥1% of 16S rRNA gene sequences within a single sample – were more commonly identified by LEfSe as potential markers for controls, though some could represent potential contaminants (Marsh et al., 2018).

### Core Microbiota

**FIGURE 4:**
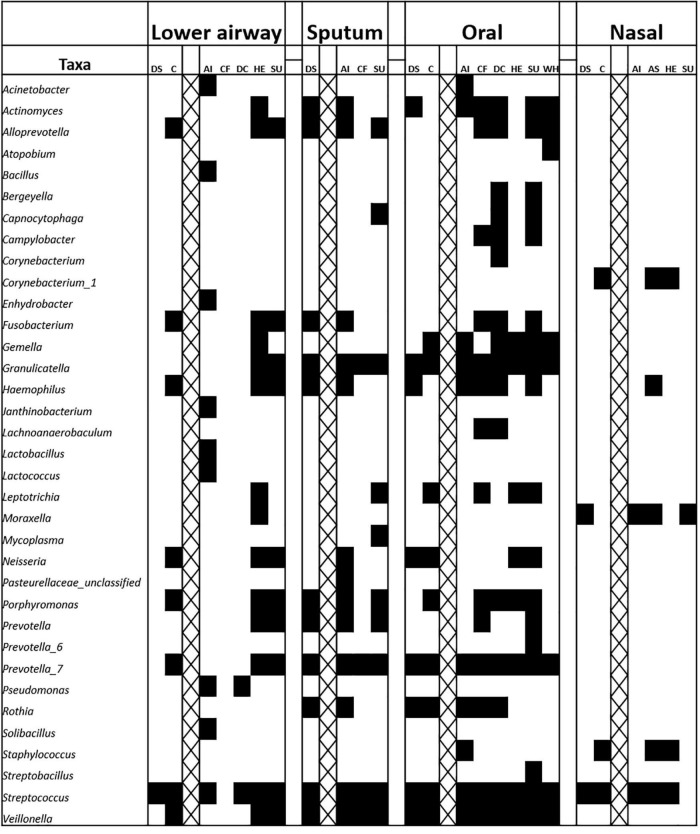
Representation of bacterial genus-level phylotypes in the core microbiota from nasal, oral, sputum, and lower airway samples. A core was defined as presence in at least 75% of samples, based on the rarefied data. An abundance filter was also applied, whereby a genus must represent ≥10% in at least one sample. Cross-hatching separates broad-level comparisons from those involving specific diagnostic groupings, within a given anatomical site. AI, acute infections; AS, asthma; C, control; CF, cystic fibrosis; DC, disease control; DS, disease (any respiratory diagnosis); HE, healthy; SU, suppurative; WH, wheezing illness.

Clinical samples often contain a wide variety of microbial taxa and identifying which, if any, of these could be relevant to disease pathology is not straightforward. Core microbiota approaches reduce complexity of microbiota analyses by focusing on only the most prevalent (and in some cases abundant) members of a bacterial community (Astudillo-García et al., 2017). Filtering data to retain only genera present in ≥75% of samples with a relative abundance of ≥10% in at least one sample for a given anatomical site revealed that members of the genus *Streptococcus* were present in the core microbiota of almost all diagnostic groups, irrespective of anatomical site (Figure 4). The genera *Prevotella*, *Haemophilus*, and *Granulicatella* were also widespread. Notably, there was no 75%-core microbiota for lower airway samples from CF patients, although LEfSe did identify *Pseudomonas* as a marker of this group. Intuitively, cores for specific diagnostic groups were typically larger than those for overall disease or control cores, potentially due to the lower number of samples required to meet the prevalence threshold and/or other factors common to samples within a given diagnostic group.

### Microbiota Homogeneity Within and Between Diagnostic Groupings

To determine the extent to which different clinical diagnostic groupings overlap or differ in terms of their microbiota, we analysed bacterial beta-diversity. Bray–Curtis dissimilarity (a common measure of beta-diversity) was similar within and between broad control vs. disease groupings regardless of anatomical site (Supplementary File A and Supplementary Figure E7). By contrast, in the finer diagnostic groupings Bray–Curtis dissimilarity was highly dependent on both anatomical site and diagnostic grouping. For instance, in nasal samples values within and between groups were similar (indicating equivalent levels of dissimilarity and overall general lack of microbiota distinctiveness for a given diagnosis). In contrast, for some oral (e.g., suppurative, wheezing illness) and lower airway (e.g., acute infections, suppurative) groupings there was greater microbiota homogeneity within compared to between groups, suggesting a more distinct microbiota associated with these diagnostic groupings. According to PERMANOVA analysis, variables contributing most to variability in the microbiota data were individual study (encompassing multiple technical factors; 12.4% of variation explained), anatomical site (2.5%) and diagnostic group (2.4%) (Supplementary File B and Supplementary Table E17).

### Detection of Lower Airway Disease Based on Microbiota Profiles

**FIGURE 5:**
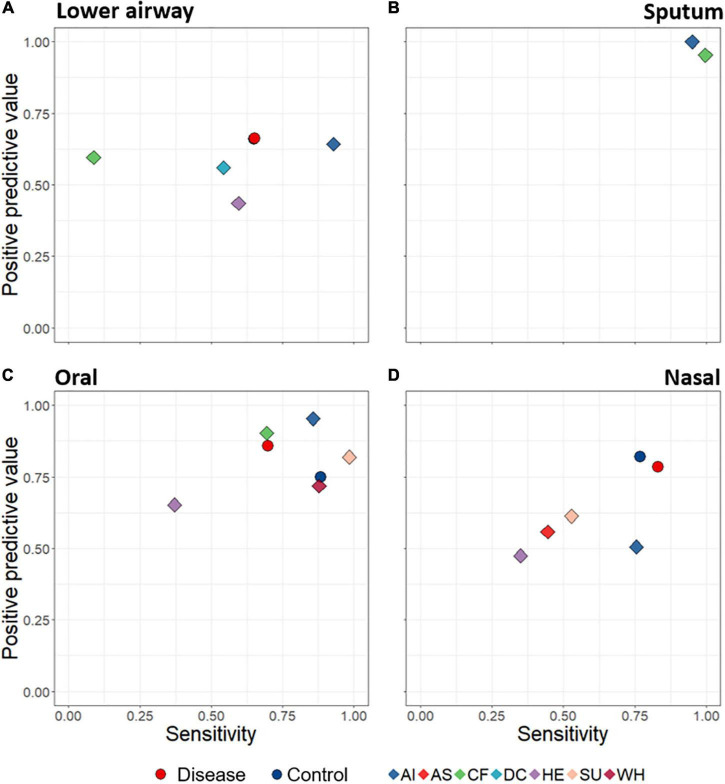
Average positive predictive value (fraction of calls of a diagnostic grouping which are correct) and sensitivity (fraction of samples within a diagnostic grouping which are correctly identified) rates of sample assignments to both broad disease level (circles) and specific diagnostic groupings (diamonds), through use of random forest machine learning. Data are displayed according to anatomical category: lower airways **(A)**, sputum **(B)**, oral **(C)**, and nasal **(D)**. Predictions were made based on rarefied data in which the numbers of samples for each diagnostic grouping were made equal. The Control symbol (blue circles) in **(D)** is hidden behind the red (Disease) circle. AI, acute infections; AS, asthma; CF, cystic fibrosis; DC, disease control; HE, healthy; SU, suppurative; WH, wheezing illness.

A key aim was to determine whether lower airway disease could be detected based on composition of upper- and/or lower-airway microbiota. We thus developed a machine learning (random forest) model to predict disease state based upon bacterial distinctiveness of different clinical diagnoses, i.e., is there a distinct microbiota “signature” that allows us to detect different diseases? Machine learning predictions were assessed according to positive predictive value (fraction of calls of a diagnostic grouping which are correct) and sensitivity (fraction of samples within a diagnostic grouping which are correctly identified) for all specific diagnostic groupings. When attempting to use microbiota data alone to predict from which diagnostic group a sample came, success varied with both diagnostic group and anatomical site (Figure 5). For example, sputum samples were particularly effective at distinguishing among diagnostic groupings (with both positive predictive value and sensitivity scoring close to 1), while oral samples also performed well. Lower airway samples were poorest overall at identifying lower airway disease, while detection success of nasal samples varied considerably.

### Sensitivity Analyses

Sensitivity analyses investigating effects of participant age, sequence data normalisation approach (rarefaction vs. GMPR), and the Wang et al. (2016) and Luna et al. (2018) studies, yielded results largely consistent with those presented above (for details see Supplementary File A).

## Discussion

Our compilation and re-analysis of 16S rRNA gene data using a uniform bioinformatic pipeline of specimens from >2500 individuals obtained from 20 distinct studies enabled testing of three important hypotheses in childhood respiratory diseases. Inclusion of multiple diseases in the same IPD meta-analysis facilitated the search for overarching microbiota patterns of health and disease. The importance of such an approach was highlighted recently for the gut, where half of all genera linked to specific diseases in a single study were in fact associated with more than one disease (Duvallet et al., 2017). Application of this approach to the respiratory microbiota yielded several novel findings with potential clinical implications.

### Airway Microbiota Diversity Differs Between Health and Disease

Observed relationships between bacterial diversity and respiratory disease are complex and vary among different conditions, clinical states and sample types (Man et al., 2017). The primary literature is inconsistent, with reports of both higher (Sakwinska et al., 2014; Cuthbertson et al., 2017) and lower (Marsh et al., 2016; Kim et al., 2017) bacterial diversity in controls compared to disease states. Our IPD meta-analysis revealed significantly lower bacterial diversity with disease in samples of nasal and lower airway origin, but the opposite trend for the oral microbiota. In contrast to diversity, overall microbiota profiles were similar for a given anatomical site, with major bacterial taxa such as *Moraxella*, *Streptococcus*, *Haemophilus*, and *Neisseria* prominent in both health and disease. What *did* sometimes differ was relative abundances of specific genera, with the ubiquitous *Streptococcus* notable for its greatly increased abundance in nasal samples from individuals with disease. Rather than a wholesale shift in microbiota composition, disease may instead manifest more as a decrease in bacterial community evenness, with one or more “bloom” taxa increasing their abundance relative to others. Antibiotic usage, while not explicitly tested here due to a lack of comparable data, may contribute to observed reductions in diversity. The most dramatic example of reduced diversity was in the lower airways of children with CF. Although the exacerbation status of these patients may contribute to this low alpha-diversity, the dataset included a mixture of clinically stable and exacerbating individuals with the original study noting no differences in diversity with exacerbation (Zemanick et al., 2017). Whilst speculative, arresting disease progression *via* microbiota preservation and/or restoration, particularly in long-term or permanent conditions such as CF in which diversity loss may be most marked, may be feasible with wider use of complementary, antibiotic-free approaches such as physiotherapy, anti-inflammatory drugs, probiotics, and vaccines as the first line of defence against disease.

### Specific and Non-specific (Overarching) Microbiota Signatures Across Diagnostic Groups

Our analyses revealed that some findings, such as *Pseudomonas* as a potential biomarker of CF in lower airway samples, were specific to an individual diagnostic group and consistent with previous literature (Emerson et al., 2002). We also saw evidence for non-specific signatures of respiratory disease. LEfSe biomarker analysis identified more putative markers for disease overall than for any single diagnostic grouping. This highlights the need for caution when comparing disease to controls for a single condition, in that one may identify apparent markers of that disease which are in fact more general markers of *multiple* respiratory diseases. Moreover, shared phylotypes in the cores of multiple diagnostic groupings, but not in control cores, provided further evidence for a non-specific disease signature. *Moraxella* within nasal samples is a standout example, being present in all disease group cores but not corresponding control cores. These findings are supported by previous research suggesting a shared core microbiota among different diseases (van der Gast et al., 2014). Caution should therefore be exercised when considering specific bacterial taxa identified *via* microbiota analyses as potentially diagnostic of particular diseases.

### Microbiota-Based Detection of Clinical Diagnoses

We used machine learning to evaluate the ability of microbiota profiles to determine clinical diagnostic categories. Sputum and to a lesser extent oral samples yielded the most promising results. Whether this reflects true biological signal or technical biases influencing the random forest model remains unclear. For example, sputum was derived from only two distinct diseases with underlying cohort differences, likely enhancing assignment capabilities beyond that attributable to the microbiota. Indeed, the fact that diagnostic groupings in this meta-analysis were largely reflective of research groups, all of which had distinct methodological characteristics, creates a largely unavoidable bias within the data and presents a challenge for the machine learning model. Such biases could explain the counter-intuitive finding that the microbiota of lower airway specimens appeared to be *least* effective for detecting lower airway disease, implying the lack of a strong disease signal in the lower airway. However, this result should be interpreted with caution and there is a need for further studies using standardised analytic methods to support or refute these preliminary findings. The previously mentioned technical differences among studies may also explain some of our findings, including that related to the lower airways, yet there was still a detectable signal of diagnostic grouping. Parallel PERMANOVA analyses identified a significant albeit minor contribution of diagnostic grouping to underlying variability within the data, even after accounting for technical factors. Ongoing validation of machine learning (using larger datasets and greater standardisation of approaches) may ultimately lead to a complementary diagnostic strategy for diagnosing paediatric lower airway disease *via* relatively non-invasive sampling and analysis of the upper airway microbiota.

### Methodological Considerations

While the meta-analysis approach is a powerful one, it does have constraints. Some combinations of diagnostic grouping and anatomical site were represented by a single study, limiting more general conclusions. Moreover, even with such a large dataset (∼2800 samples from >2600 individuals) power is quickly lost when attempting to split the data into specific categories. Applying a uniform bioinformatics pipeline removes a potentially substantial source of variation, but clinical (e.g., exacerbation vs. clinical stability) and technical factors (e.g., sampling, DNA extraction) will still contribute to variability within the microbiota data where there is methodological heterogeneity between studies. Indeed, the high explanatory power assigned to individual study by PERMANOVA corroborates the pervasive influence of laboratory-specific approaches and highlights the benefits of a more standardised, cross-laboratory approach. The application of batch correlation tools, particularly where both case and control data are available (Gibbons et al., 2018), also warrants further investigation in an attempt to account for some of these factors. Another useful aspect would be the routine inclusion of both negative technical controls (to detect contaminants) and quantitative approaches such as real-time PCR or droplet digital PCR (to estimate bacterial load). The limited taxonomic resolution of genus-level phylotypes is also noteworthy. The phylotype approach was necessary due to the different 16S rRNA gene regions sequenced, preventing application of operational taxonomic unit or amplicon sequence variant approaches (Callahan et al., 2017). Species- or strain-level differences are likely to be important clinically, and a future focus on deciphering such interactions is warranted. *Streptococcus* provides a salient example: this genus was prevalent and abundant throughout the assembled dataset, but our analyses based on short-read 16S rRNA gene sequences cannot determine whether this was a single species (e.g., pneumococcus) or, more likely, many different species. This is an inherent limitation of 16S rRNA approaches more generally, due to the conserved nature of this gene and the short-read sequences generated by amplicon sequencing techniques. Additionally, while we relied on LEfSe for detecting differential abundance between controls, disease and different diagnostic groups, this is but one of many techniques for identifying differential taxa. Recent comparative studies of various differential abundance approaches have highlighted both considerable variation in outcomes when different techniques are applied and the lack of a clear-cut candidate for the best available tool at present (Nearing et al., 2021; Wallen, 2021). Finally, in our analysis we only considered bacterial members of the respiratory microbiota, whereas viruses and fungi also likely play key roles within the respiratory tract (Wylie, 2017; Cox et al., 2019; Cuthbertson et al., 2020).

## Conclusion

Despite some limitations, our IPD meta-analysis offered key advantages not available through other approaches. Ethical and logistical considerations associated with sampling the airway microbiota contribute to a paucity of case-control studies (only 60% of included studies contained both cases and controls), constraining the ability of individual studies to explicitly compare health and disease. While this complicates interpretation of broader patterns within the airway microbiota, especially compared with the more accessible microbial communities sampled from human faeces (Duvallet et al., 2017), this meta-analysis enabled disease samples from studies lacking controls to be compared to controls from other studies. Additionally, using sensitivity analyses and the twofold (rarefaction and GMPR) normalisation approach, we were able to evaluate the association of various factors presumed to affect microbiota profiles. Re-analysis of multiple studies also enabled comparison of many more diseases than would be feasible in a single study. While airway bacterial diversity differed between health and disease, other differences were more subtle with a combination of non-specific and anatomical site-dependent contributions to microbiota signatures of any specific diagnostic grouping. Identifying such factors *via* meta-analyses is a further step toward development of novel treatments aimed at rebalancing the airway microbiota, in a manner analogous to faecal transplants and other interventions focused on the gut (Ali and Sweeney, 2020). Moreover, our analysis provides a platform to build future prospective studies where diagnostic categories are uniform.

## Data Availability Statement

The data analysed in this study is subject to the following licences/restrictions: we have not made any new datasets available through this study, as data availability is subject to the original studies and their particular ethics agreements. Requests to access these datasets should be directed to the corresponding authors of the original studies.

## Ethics Statement

Ethical approval was not provided for this study on human participants because separate ethics approvals had been obtained for each original study and the University of Auckland Human Participants Ethics Committee deemed that re-use of these published data did not require additional approval. Written informed consent for participation was not provided by the participants’ legal guardians/next of kin because separate ethics approvals had been obtained for each original study and the University of Auckland Human Participants Ethics Committee deemed that re-use of these published data did not require additional approval.

## Author Contributions

DB: research design, data collation, bioinformatic analysis, and co-wrote manuscript. DW: bioinformatic pipeline development, data interpretation, and contributed to manuscript writing and editing. MT: conceived study, research design, data interpretation, and co-wrote manuscript. RM, CC, AC, and NP: research design, facilitated access to original data, data interpretation, and contributed to manuscript writing and editing. JH, KH, JM, GO’T, and CvdG: facilitated access to original data, data interpretation, and contributed to manuscript writing and editing. PC, WC, LC, WD, ME, AG, LH, S-JH, LJ, MK, B-SK, YK, SL, AM, LP, MP-L, MPe, MPi, OR, LR, OS, PS, BW, HY, EZ, and YZ: facilitated access to original data, data interpretation, and contributed to manuscript editing. All authors contributed to the article and approved the submitted version.

## Conflict of Interest

MPi reports personal fees from Mérieux Université, grants from Abacus Diagnostica, outside the submitted work. AM reports grants and personal fees from Janssen, personal fees from Merck, personal fees from Sanofi-Pasteur, personal fees from Roche, outside the submitted work. EZ reports grants and personal fees from Cystic Fibrosis Foundation, outside the submitted work. OS is an employee of Nestlé Research – Societé des Produits Nestlé S.A. The remaining authors declare that the research was conducted in the absence of any commercial or financial relationships that could be construed as a potential conflict of interest.

## Publisher’s Note

All claims expressed in this article are solely those of the authors and do not necessarily represent those of their affiliated organizations, or those of the publisher, the editors and the reviewers. Any product that may be evaluated in this article, or claim that may be made by its manufacturer, is not guaranteed or endorsed by the publisher.
