# Supplementary material for: Bacterial Signatures of Paediatric Respiratory Disease: An Individual Participant Data Meta-Analysis
Source: Front Microbiol. 2021 Dec 23;12:711134. doi: 10.3389/fmicb.2021.711134 (PMC8733647; doi:10.3389/fmicb.2021.711134)
Supplement: Supplementary file 1 [file Table_1.DOCX]

**Bacterial signatures of paediatric respiratory disease: an individual participant data meta-analysis**

David TJ Broderick, David W Waite, Robyn L Marsh, Carlos A Camargo Jr, Paul Cardenas, Anne B Chang, William OC Cookson, Leah Cuthbertson, Wenkui Dai, Mark L Everard, Alain Gervaix, J Kirk Harris, Kohei Hasegawa, Lucas R Hoffman, Soo-Jong Hong, Laurence Josset, Matthew S Kelly, Bong-Soo Kim, Yong Kong, Shuai C Li, Jonathan M Mansbach, Asuncion Mejias, George A O’Toole, Laura Paalanen, Marcos Perez-Losada, Melinda M Pettigrew, Maxime Pichon, Octavio Ramilo, Lasse Ruokolainen, Olga Sakwinska, Patrick C Seed, Christopher J van der Gast, Brandie D Wagner, Hana Yi, Edith T Zemanick, Yuejie Zheng, Naveen Pillarisetti, Michael W Taylor ^*^

Supplementary File A

* Corresponding author:

Mike Taylor

School of Biological Sciences

University of Auckland

Private Bag 92019

Auckland 1142

New Zealand

Phone: +64 9 9232280

E-mail: [mw.taylor@auckland.ac.nz](mailto:mw.taylor@auckland.ac.nz)

**Table of contents (Supplementary File A)**

| Detailed description of analysis approach | 3 |
| --- | --- |
| Detailed description of sensitivity analysis | 5 |
| Funders of original studies | 7 |
| Full list of search terms used in literature search | 10 |
| Full exclusion and inclusion criteria | 11 |
| Sequenced 16S rRNA gene regions from each study (Figure E1) | 12 |
| Non-metric multidimensional scaling (nMDS) plots (Figures E2 – E6) | 13 |
| Bacterial beta-diversity plots (Figure E7) | 18 |
| Rank-abundance plots: rarefaction vs GMPR-normalized datasets (Figure E8) | 19 |
| 16S rRNA gene sequence depths for included studies (Figure E9) | 20 |
| Machine learning results based on GMPR-normalized data (Figure E10) | 21 |
| Code for bioinformatics pipeline | 22 |
| Code for machine-learning analyses | 24 |
| Online supplement references | 26 |

**Detailed description of analysis approach**

**Bioinformatic processing**

The analysis pipeline utilised USEARCH (v.11.0667) (Edgar 2010) for quality filtering and, where applicable, merging of paired-end reads, after which reference gene alignment and chimera removal (chimera.uchime) (Edgar et al., 2011) were performed using mothur (v1.38.1) (Schloss et al., 2009) Taxonomic classification (classify.otu) was performed in mothur using the SILVA SSU database (v132) as reference (Quast et al., 2012). Sequences assigned to non-bacterial lineages or that could not be identified to genus level were removed. Reprocessing of the data led to removal of 557 samples not containing any reads which could be identified at genus level through our uniform pipeline. Where possible, to gain sub-genus classification, the database identifier was used to further discriminate within groups, for example *Prevotella*_7 and *Prevotella*_3.

**Data normalisation**

To evaluate different data normalisation approaches, we attempted to recapitulate the original claims made by a subset of studies. The five normalisation approaches assessed were: rarefaction, centre-log transformation, relative abundance, Z-transformation and geometric mean of pairwise ratios (GMPR). This was used to identify the most appropriate data normalisation approach and any influence that our processing pipeline may have on conclusions obtained from the respective datasets. Due to the very large range of sequencing depths present within our overall dataset we addressed concerns about applying a common rarefaction threshold by evaluating test claims at both a common depth (1000 sequence reads per sample) and at a unique sequence depth tailored for each individual study. Both of these rarefaction thresholds allowed us to largely recapitulate most findings from the original papers, therefore a consistent threshold of 1000 sequences per sample was applied for subsequent analyses. GMPR performed best among the non-rarefaction approaches and was subsequently used for comparison with rarefaction. For consistency between normalisation approaches, samples with <1000 sequences (348 in total) were removed prior to normalisation.

**Data cleaning**

Following normalisation, a final data cleaning step was applied to avoid bias due to over-representation of a particular bacterial community (i.e. pseudoreplication). This was necessary because several studies sampled multiple anatomical sites within each participant. In order to mitigate data loss, the data were split into four broad anatomical sites with distinct physicochemical features (nasal, oral, sputum, lower airways (bronchoalveolar lavage or bronchial brushings)) (Marsh et al., 2016, Man et al., 2017, Ronchetti et al., 2018), but cross-site comparisons were not performed. Where an individual had samples from more than one anatomical site, all samples were retained. However, if an individual had more than one sample from the same broad anatomical site (e.g. anterior nares and nasopharyngeal swabs), only one was retained. Where samples were identical in nature (i.e. technical replicates), the sample which had the highest sequencing depth prior to normalisation was selected. There were two examples where samples were non-identical in nature, namely Sakwinska *et al.* (2014), and Luna *et al.* (2018). In the Sakwinska paper, two distinct gene regions were sequenced for each sample (V1-V2 and V4). We used the V4 data for our analyses as this had the higher average sequencing depth prior to normalisation. In the Luna paper, samples were collected from both the nasopharynx and anterior nares of each patient. Here, we retained only the nasopharyngeal samples in the main dataset as the majority of samples within the broader nasal anatomical site from other studies were nasopharyngeal in origin. It is worth noting that this did create a conflict in which the majority of the disease samples were obtained from the nasopharynx, while control samples were primarily from the anterior nares: this conflict was addressed in a sensitivity analysis as described below. Ultimately, 2789 samples (from 2624 individuals) were retained for subsequent analysis.

**Statistical analyses**

Statistical analyses were performed in R (v3.6.2) (R Core Team, 2019), primarily using the vegan package for calculating bacterial diversity statistics and ggplot2 (Wickham, 2016) for generating plots.

The alpha diversity evenness metrics Shannon and Gini-Simpson were calculated using the diversity function in vegan, while the richness metrics observed phylotypes (recorded as S.Obs) and ACE were calculated by the EstimateR function in vegan. While not shown in the paper, we also calculated the Chao1 statistic, which returned results consistent with the other richness metrics. For calculation of alpha- and beta-diversity statistics, only rarefied data were used, consistent with recommendations of the GMPR developers (Chen et al., 2018). Differences in alpha diversity were assessed using both parametric and non-parametric approaches; for differences between controls and disease a Bonferroni-corrected t-test (stats::t.test) was used for the former, while the non-parametric Wilcoxon rank-sum tests (stats::wilcox.test) was also applied. Differences among diagnostic groupings were also tested using both parametric (analysis of variance (stats::aov) followed by Tukey’s Honestly Significant Difference test (stats::TukeyHSD)) and non-parametric (Kruskal-Wallis test followed by a Dunn’s test (FSA::dunnTest)) approaches. The results of the parametric and non-parametric approaches were highly similar.

Beta-diversity was estimated using Bray-Curtis distance and calculated using the vegdist function in vegan. Bray-Curtis distances were also used to generate non-metric multidimensional scaling (nMDS) plots, which supported splitting of the data by anatomical site. The nMDS analyses also identified the Wang *et al.* (2016) dataset as anomalous; based on this observation we undertook a sensitivity analysis due to the distinct clustering of those data. To determine the proportion of variation in the microbiota data explained by various factors, we performed permutational analysis of variance (PERMANOVA) using the adonis function in R. This function was performed using a Bray-Curtis distance matrix.

Figure 3 in the main manuscript was created by determining the average relative 16S rRNA gene sequence abundance across all samples for control and disease at each anatomical site. As we had to assign a reference group for the rankings shown in this plot (in this case we used Control samples), it was possible that within the non-reference (i.e. Disease) group there existed a genus-level phylotype which was highly abundant but not shown in the plot. To control for this, we repeated the analyses with Disease rather than Control samples as the reference group for assigning ranking, but the most abundant taxa remained highly similar.

Genus-level phylotypes which differed significantly between diagnostic groupings were identified using linear discriminant analysis effect size (LEfSe) using the Galaxy platform (Segata et al., 2011).

**Core microbiota and machine learning analyses**

We identified the core microbiota for specific anatomical sites and diagnostic groupings by applying a prevalence threshold to identify phylotypes occurring in ≥75% of the respective samples. Only phylotypes that occurred at ≥10% relative abundance in at least one sample are shown in Figure 4 in the main manuscript.

To determine whether diagnostic groups could be identified based only on microbiota composition, machine learning trials were performed on rarefied data in python (Pedregosa et al., 2011). For this, 60% of samples from a given anatomical site were selected at random to use as training data, with the remaining 40% used for validation. Of five different initial approaches (random forest; neural network; support vector machine (SVM): linear; SVM: radial bias function; SVM: polynomial), random forest was most successful across the majority of trials. Success in this context was determined by the accuracy value (the fraction of correct calls over all calls) (Sokolova et al., 2006). The random forest approach was then applied independently to both the rarefied and GMPR-normalized datasets using the training and validation strategy outlined above. In addition, for both normalisation methods we generated a sample dataset in which the contribution of different diagnostic groupings was set as equivalent to account for differences in sample numbers. These final machine learning approaches were assessed for both their positive predictive value (defined as the fraction of calls of a diagnostic grouping which are correct) and sensitivity (defined as the fraction of samples within a diagnostic grouping which are correctly identified) for all specific diagnostic groupings.

**Detailed description of sensitivity analysis**

**Introduction**

Sensitivity analyses aim to test the robustness of meta-analysis findings to the decisions used to generate them (Deeks et al., 2019). For example, if one was to analyse how smoking might increase the risk of lung cancer, it may be worthwhile to isolate and consider the effect of family history, as this is also a risk factor for lung cancer (Schwartz et al., 1996). To do this in a sensitivity analysis you could re-run the analyses by isolating different age groups to determine if the risk was similar. Many factors are known to, or likely to, influence the paediatric respiratory microbiota. For some of these factors, where consistent methods of reporting meant that sufficient data were available, we were able to conduct a sensitivity analysis to consider their impact. It is important to note that we were not attempting to determine the influence of factors such as age on the microbiota *per se*; rather, we sought to determine whether our overall conclusions changed upon accounting for these various factors.

Factors which were considered in these sensitivity analyses included:

Age: This was treated as a categorical variable in the following groups:

Birth to <1 year old

1 to <2 years old

2 to <5 years old

5 to <18 years old

Study: Two studies had the potential to skew interpretation of the data:

Wang *et. al.*, 2016: This study was considered as it clustered independently in ordination plots (including nMDS) based on both the overall dataset and the lower airway data.

Luna *et. al.*, 2018: This study was considered as it made up a large component of nasal samples for acute infections (and indeed a substantial proportion of the entire dataset), so it was necessary to check that our findings were not simply replicating those from this single study.

Anatomical site: While we did differentiate among broad anatomical sites, in the nasal samples the control subjects were predominately anterior nare samples, while those with disease were predominately taken from the nasopharynx. The study of Luna *et al.* allowed us to test for these differences due to their paired sample collection.

Data normalisation approach: In the main paper, analyses shown were based on rarefying sequence data to 1000 sequences/sample. To determine the influence of data normalisation approach on our conclusions, we also conducted all analyses (excluding those involving alpha- and beta-diversity metrics) using the geometric mean of pairwise ratio (GMPR) approach. This size factor-based approach is less affected by data compositionality than rarefaction.

**Methods**

We performed sensitivity analyses in two different ways, which can be broadly described as “removal” and “isolation”. *Removal* involved the elimination of all samples from a particular category (e.g. a specific age group or study), then the analyses were repeated. *Isolation* involved retention only of those samples pertaining to the single category in question, again followed by re-analysis. The aim of both approaches was to determine whether our initial overall findings could be recapitulated. While we could satisfactorily perform these analyses with microbiota metrics such as alpha- and beta-diversity, core microbiota (and to an extent LEfSe) analyses were not conducive to this approach, particularly when considering age category and different diagnostic groupings, due to the drastic subsetting of data involved. For example, isolation of a particular age group may also lead to concomitant isolation of a single study and/or diagnostic grouping, as well as a potential marked reduction in statistical power.

Using age as an example, all children <1 year of age were removed from the relevant datasets in the removal approach, while in the isolation approach *only* samples from that demographic were considered. For consideration of the Wang *et. al.* (2016) and Luna *et al.* (2018) studies, only the removal approach was used as isolation would achieve nothing other than duplication of the original study.

For addressing the anatomical site question outlined above, a slightly different approach was taken in which the nasopharyngeal samples from Luna *et al.* (2018) were exchanged for the anterior samples from the same study.

GMPR was carried out by using the source code supplied with the original paper by Chen *et al.* (2018).

The outcomes of our sensitivity analyses are described briefly below.

**Results**

The nature of the sensitivity analyses was such that differences related to removal or isolation of a given factor could not be compared by a single test statistic. We therefore report below the effect (if any) that adjusting the dataset for the analysed factors had on our major conclusions. Further details are available from the authors upon request.

The ranking of which factors explained the largest amount of variation within the microbiota data (as evaluated by PERMANOVA) was broadly consistent across the various analyses. For example, individual study (and the inherent technical factors associated with it) consistently accounted for the largest amount of microbiota variation, irrespective of which factors were examined (i.e. which data subsets were removed or isolated). Other specific outcomes are described below.

Age: Overall, age categories did not have a substantial effect on the outcomes of our analyses. Major trends such as reduced alpha-diversity in disease (in samples of nasal and lower airway origin) were conserved regardless of which age category was included or excluded.

Study: As with age, individual removal of the two aforementioned studies (Wang *et al.* 2016, Luna *et al.* 2018) had negligible impact on our key conclusions. For example, differences in alpha-diversity between health and disease remained statistically significant upon removal of either study.

Anatomical site: Our overall conclusions were consistent irrespective of whether anterior nares or nasopharyngeal samples were included in the analysis. Trends in both alpha- and beta-diversity were retained, however there were some differences in the identification of biomarkers via LEfSe. For example, we reported in the main manuscript (which included only the nasopharyngeal samples from the Luna paper) that *Haemophilus* and *Streptococcus* were identified by LEfSe as potential markers of disease, with *Corynebacterium_1*, *Staphylococcus* and *Dolosigranulum* associated with controls in the nasal samples. However, when the anterior nares rather than nasopharyngeal were included from the Luna dataset, *Streptococcus*, *Corynebacterium_1* and *Dolosigranulum* remained as markers, but *Haemophilus* and *Staphylococcus* did not. We were able to conduct LEfSe analyses for the anatomical site sensitivity analysis as overall samples were maintained at a sufficiently high level.

Data normalisation approach: The results derived from GMPR analyses were mostly similar to those generated with rarefied data, however the ranked abundance of certain taxonomic groups did differ. This was also reflected in the LEfSe analysis where, for instance, differences in the relative abundance of *Corynebacterium_1*, *Staphylococcus* and *Streptococcus* with health status in nasal samples were maintained, whereas *Haemophilus* and *Dolosigranulum* were no longer identified as significantly differentially abundant following GMPR. Additionally, core microbiotas generated by using GMPR-normalized data were generally larger, potentially due to better retention of rarer taxa which may be removed by rarefaction.

**Funders of original studies**

1. **van der Gast *et al.*, 2014**

Supported by: Cystic Fibrosis Foundation grant HOFFMA07P0, American Thoracic Society grant CF-07-003, National Institutes of Health grant K02HL105543, the United Kingdom Natural Environment Research Council grant NE/H019456/1, and Australia’s National Health and Medical Research Council grants 1042601, 1040830, 1,019,834 and 1,034,703.

1. **Kelly *et al.*, 2017**

Supported by: An Early Career Award from the Thrasher Research Fund, by Children’s Hospital of Philadelphia and Pincus Family Foundation, and core services from the Penn Center for AIDS Research, a National Institutes of Health (NIH)-funded program (P30-AI045008). A CIPHER grant from the International AIDS Society, supported by ViiV Healthcare. Financial support from the NIH through the Duke Center for AIDS Research (P30-AI064518). NIH T32 training grants (5T32-HD060558-04, 5T32-HD043029-13). A Hamilton Health Sciences Early Career Award.

**3. Hampton *et al.*, 2014**

Supported by: The Flatley Foundation of Boston, National Institutes of Health (NIH) grants P20 GM103413-10 and R01 HL074175-09, a Cystic Fibrosis Foundation Research Development Program grant (STANTO07R0), Cystic Fibrosis Foundation Research Development Program grant R025-CR07, NIH grants R01 HL068927-09 and R01 DK44003, Cystic Fibrosis Foundation grant CUTTIN06P0, NIH grant R01 AI091699 and NIH grant 4UH3DK083993.

**4. Marsh *et al.*, 2016**

Supported by: The Australian National Health and Medical Research Council (NHMRC) Fellowships 1034703, 1024175, 545216 and 1088733. The NHMRC Centre for Research Excellence in Respiratory Health for Aboriginal and Torres Strait Islander children (1040830) and the Channel 7 Children’s Research Foundation (Project Grant 12500).

**5. Cardenas *et al.*, 2012**

Supported by: The Wellcome Trust (grant number, 088862/Z/09/Z).

**6. Pettigrew *et al.*, 2016**

Supported by: The National Institutes of Health (grant R21DC011667 to MMP), Centers for Disease Control (grant U18 IP-000489 to JAM), and American Lebanese Syrian Associated Charities.

**7. Zemanick *et al.*, 2017**

Supported by: The Cystic Fibrosis Foundation grant ZEMANI11A0, National Institutes of Health grant K23HL114883 and NIH/NCATS Colorado CTSA grant UL1 TR001082.

**8. Perez-Losada *et al.*, 2016**

Supported by: The GWU Colonial One High Performance Computing Cluster.

**9. Zemanick *et al.*, 2015**

Supported by: The Cystic Fibrosis Foundation grant ZEMANI07DO, National Institutes of Health grant K23HL114883, and National Institutes of Health/National Center for Advancing Translational Sciences Colorado CTSI grant UL1 TR001082.

**10. Yi *et al.*, 2014**

Supported by: Research funds (2011-E43000-00, 2012-E43001-00, and 2013-E43001-00) of the Korea Centers for Disease Control and Prevention.

**11. Luna *et al.*, 2018**

Supported by: The grants U01 AI-087881, R01 AI-114552, R01 AI-108588, and UG3 OD-023253 from the National Institutes of Health.

**12. Williamson *et al.*, 2017**

The authors received no funding for this work.

**13. de Steenhuijsen Piters *et al.*, 2016**

Supported by: The Netherlands Organization for Scientific Research through NWO-VIDI grant 91715359 and ZonMW grant 91209010, and Wilhelmina Children’s Hospital intramural funds (D.B.); the National Institute of Allergy and Infectious Diseases grants AI089987 and AI112524 (O.R. and A.M.); Nationwide Children’s Hospital intramural funds grant 299814 (A.M.); and the European Society for Pediatric Infectious Diseases (ESPID Fellowship Award), the Finnish Medical Foundation, the Foundation for Pediatric Research, and Maud Kuistila Memorial Foundation (S.H.).

**14. Lu *et al.*, 2017**

Supported by: The Key Medical Disciplines Building Project of Shenzhen (201506053), Key Medical Disciplines Building Project of Shenzhen (SZXJ2017005), Guangdong Medical Research Fund (A2016501), Sanming Project of Medicine in Shenzhen (2016029), Shenzhen public service platform for clinical drug trials (20151964), and Shenzhen Science and Technology Project (JCYJ20170303155012371).

**15. Wang *et al.*, 2016**

Supported by: The Key Medical Disciplines Building Project of Shenzhen (201506053).

**16. Sakwinska *et al.*, 2014**

Supported by: The Nestlé Research Center.

**17. Cuthbertson *et al.*, 2017**

Supported by: The Wellcome Trust under WT097117 and WT096964. The NIHR Respiratory Disease Biomedical Research Unit at the Royal Brompton and Harefield NHS Foundation Trust and Imperial College London and the Sheffield Children's Hospital Charity Research Fund.

**18. Langevin *et al.*, 2017**

Supported by: Federal funds from the National Institute of Allergy and Infectious Diseases, National Institutes of Health and the Department of Health and Human Services under Centers of Excellence for Influenza Research and Surveillance (CEIRS) contract no. HHSN272201400005C.

**19. Ruokolainen *et al.*, 2017**

Supported by: Academy of Finland, the European Research Council, European Union’s Seventh Framework Programme (MeDALL), Helsinki University Hospital, Jane and Aatos Erkko Foundation and Juselius Foundation.

**20. Pillarisetti *et al.*, 2019**

Supported by: The Athlae Lyon Starship Research Trust, A+ Trust, Asser Trust and Starship Foundation.

**21. Kim *et al.*, 2017**

Supported by: A grant (2015-ER6604-00) from the Research of Korea Centers for Disease Control and Prevention.

**Full list of search terms used in literature search**

“Paediatric Respiratory Microbiota”

“Paediatric Respiratory Microbiome”

“Pediatric Respiratory Microbiome”

“Pediatric Respiratory Microbiota”

“Bronchiolitis Microbiota”

“Bronchiolitis Microbiome”

“Bronchiolitis Microbiome”

“Pneumonia Microbiome”

“Pneumonia Microbiota”

“Respiratory Syncytial Virus Microbiota”

“Respiratory Syncytial Virus Microbiome”

“Respiratory Infections Microbiome”

“Respiratory Infections Microbiota”

“Protracted Bacterial Bronchitis Microbiome”

“Protracted Bacterial Bronchitis Microbiota”

“Persistent Bacterial Bronchitis Microbiome”

“Persistent Bacterial Bronchitis Microbiota”

“Cystic Fibrosis Microbiome”

“Cystic Fibrosis Microbiota”

“Bronchiectasis Microbiome”

“Bronchiectasis Microbiota”

“Asthma Microbiota”

“Asthma Microbiome”

“CSLD Microbiome”

“CSLD Microbiota”

**Full list of exclusion and inclusion criteria**

The following criteria were used to select studies for inclusion in the meta-analysis:

- Only studies which were cross-sectional analyses (limited to one time point in the publication, even if it is part of a longitudinal study) were included.
- Only studies which investigated the following diseases were retained:
  - - bronchiolitis
    - bronchiectasis
    - cystic fibrosis
    - asthma/wheeze
    - protracted/persistent bacterial bronchitis
    - pneumonia (not ventilator associated)
    - respiratory syncytial virus infections
    - acute lower respiratory infection/upper respiratory infection
    - chronic suppurative lung disease (CSLD)
- Studies which contained next-generation 16S rRNA gene sequence data from human respiratory tract samples of fewer than 11 individuals with disease under the age of 18 were excluded.
- Studies which did not have an English version were excluded.
- Studies which focused on lung transplants or those individuals undergoing mechanical ventilation were excluded.

Cardenas 2012

Cuthbertson 2017

de Steenhuijsen Piters 2016

Hampton 2014

Kelly 2017

Lu 2017

Luna 2018

Marsh 2016

Perez-Losada 2016

Pettigrew 2016

Pillarisetti 2018

Ruokolainen 2017

Sakwinska 2014*

van der Gast 2014

Wang 2016

Williamson 2017

Yi 2014

Zemanick 2015

Zemanick 2017

1500

0

200

100

600

500

400

300

1000

900

800

700

1100

1400

1300

1200

V4

V5

V6

V7

V8

V2

V3

V1

V9

Kim 2018

**Figure E1: Sequenced 16S rRNA gene regions from each study included in the final dataset.** * The study of Sakwinska *et al.* (2014) sequenced two distinct gene regions, but only one (with the greater number of samples passing quality control) was considered in the meta-analysis.


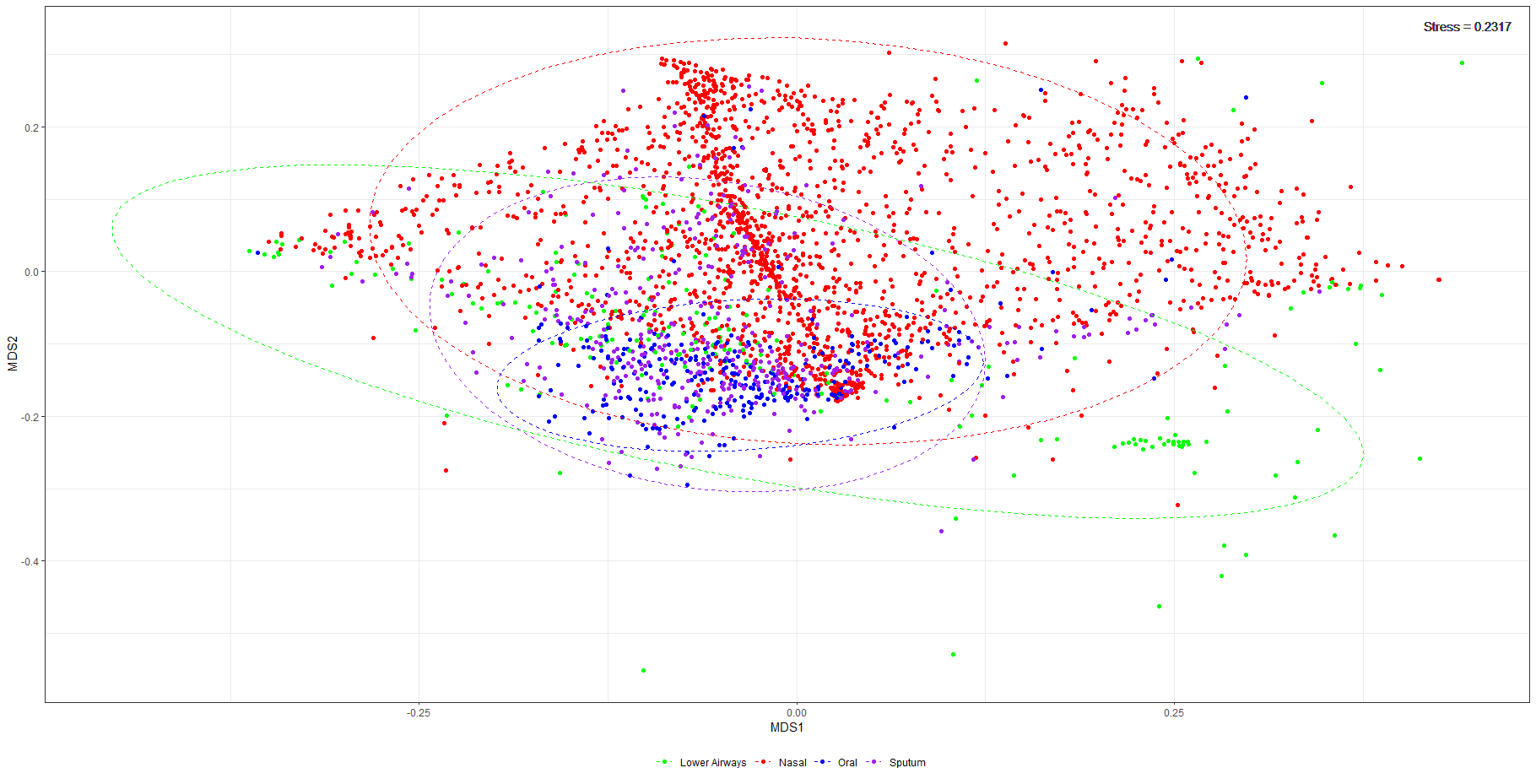

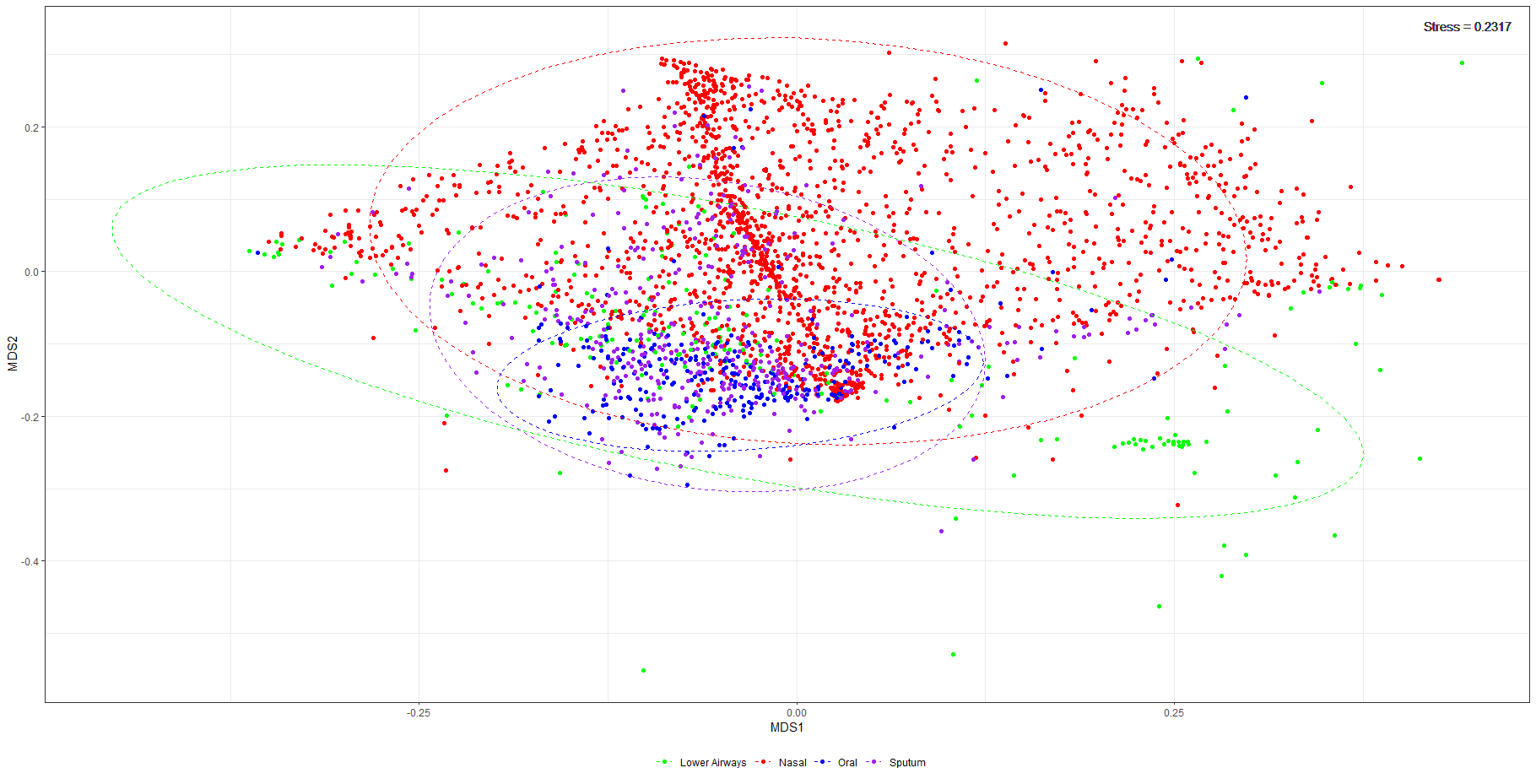


**Figure E2: nMDS plot showing the level of separation of anatomical groupings across the entire dataset (n=2789).**


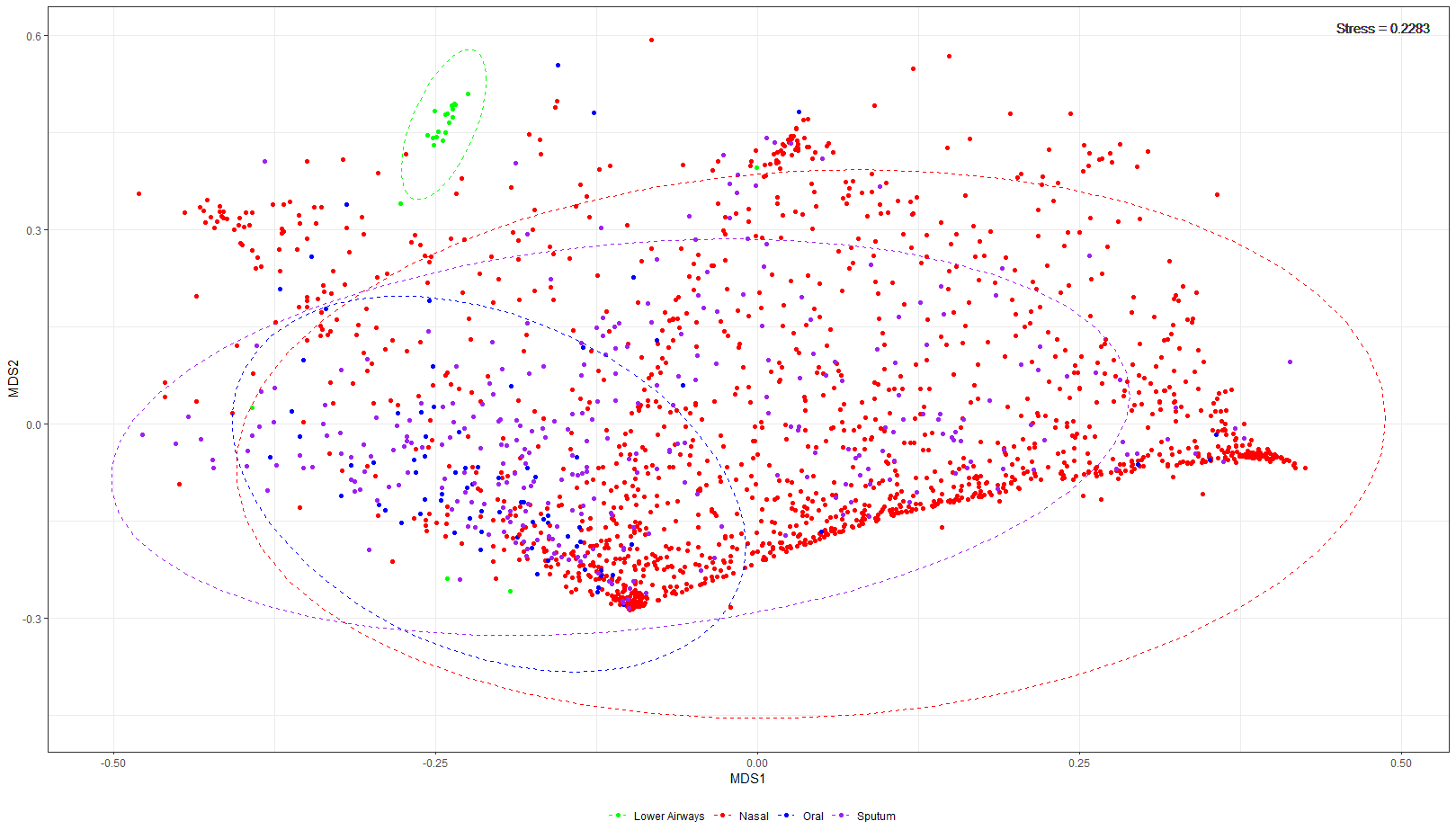

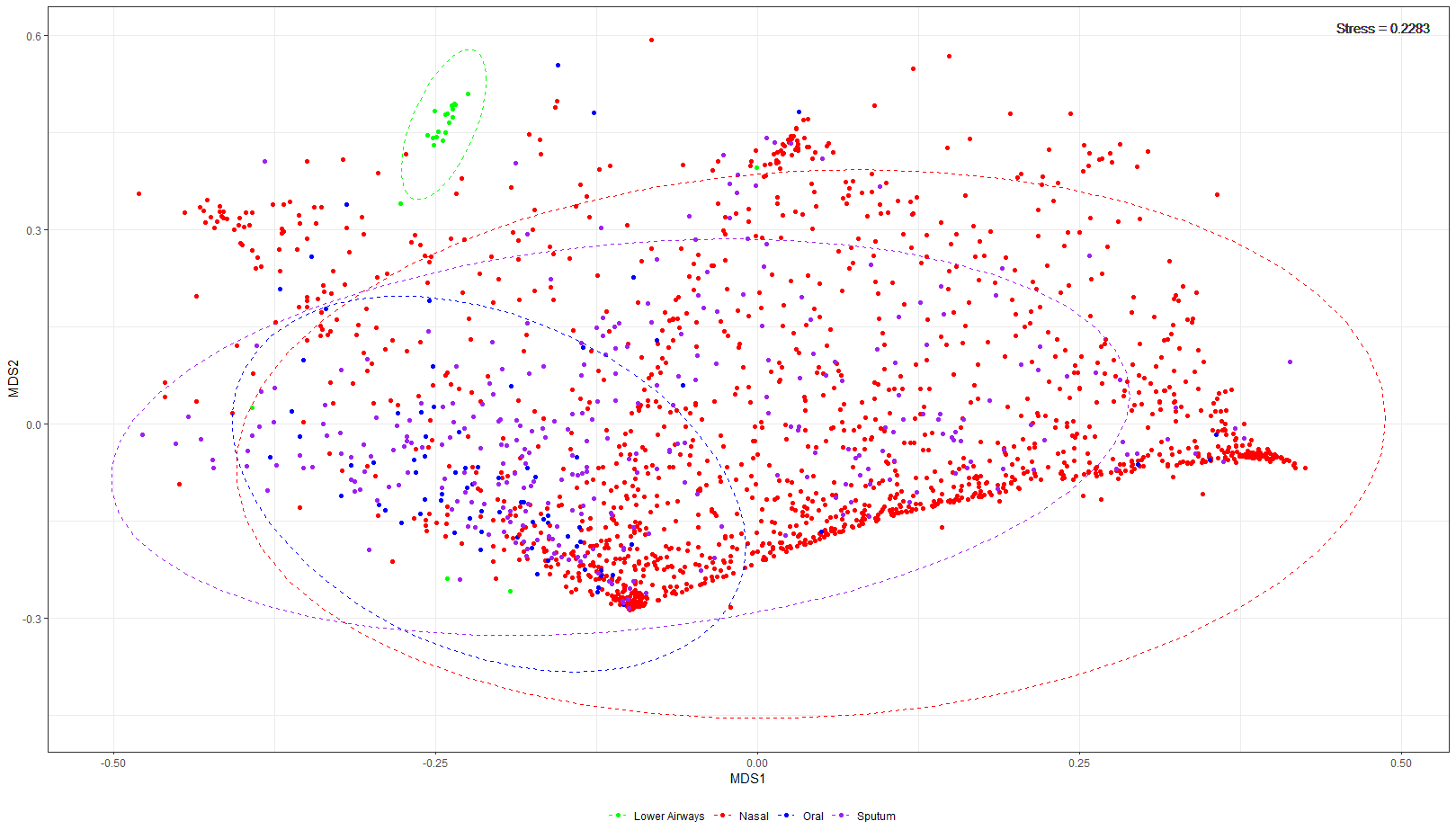


**Figure E3: nMDS plots showing the level of separation of anatomical groupings when only samples from the acute infections (n=1866) diagnostic group are considered.**


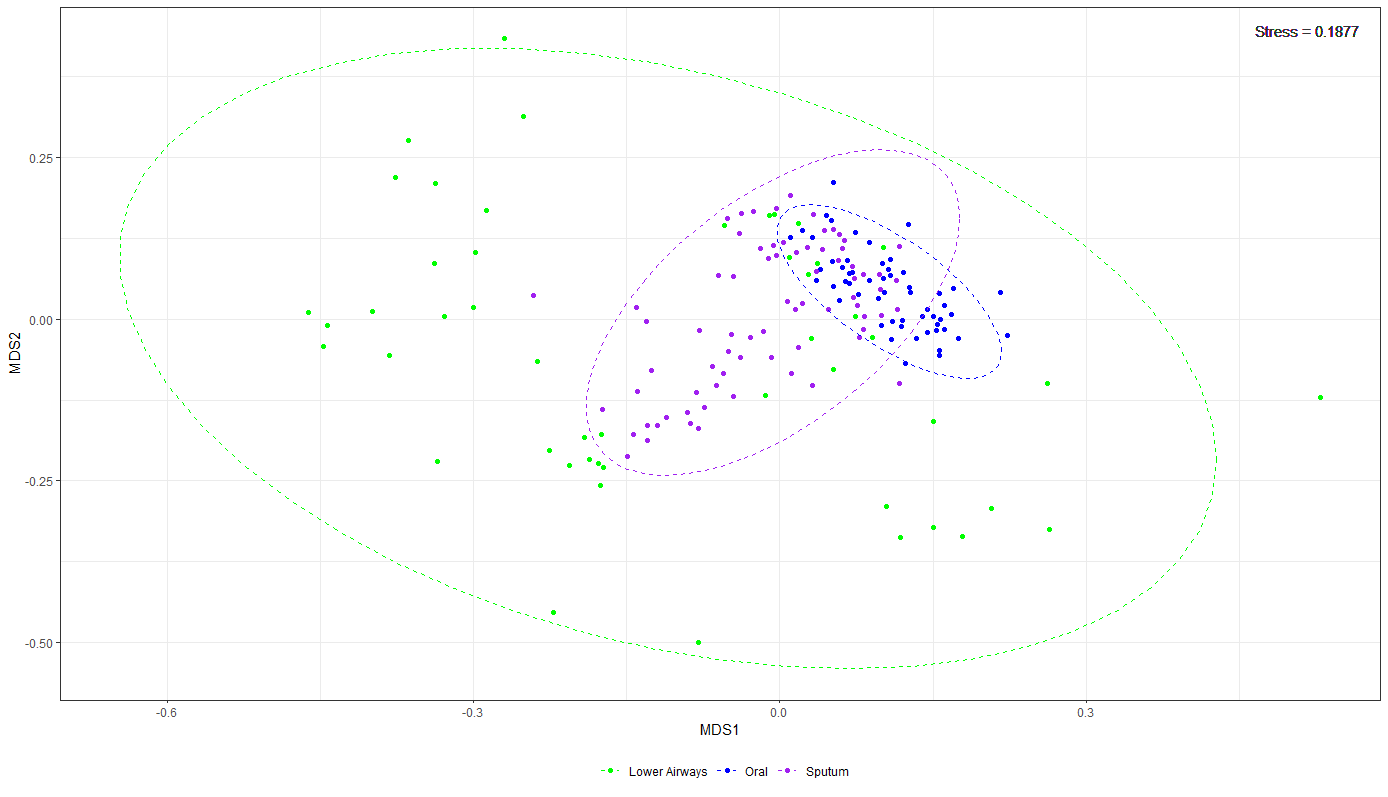

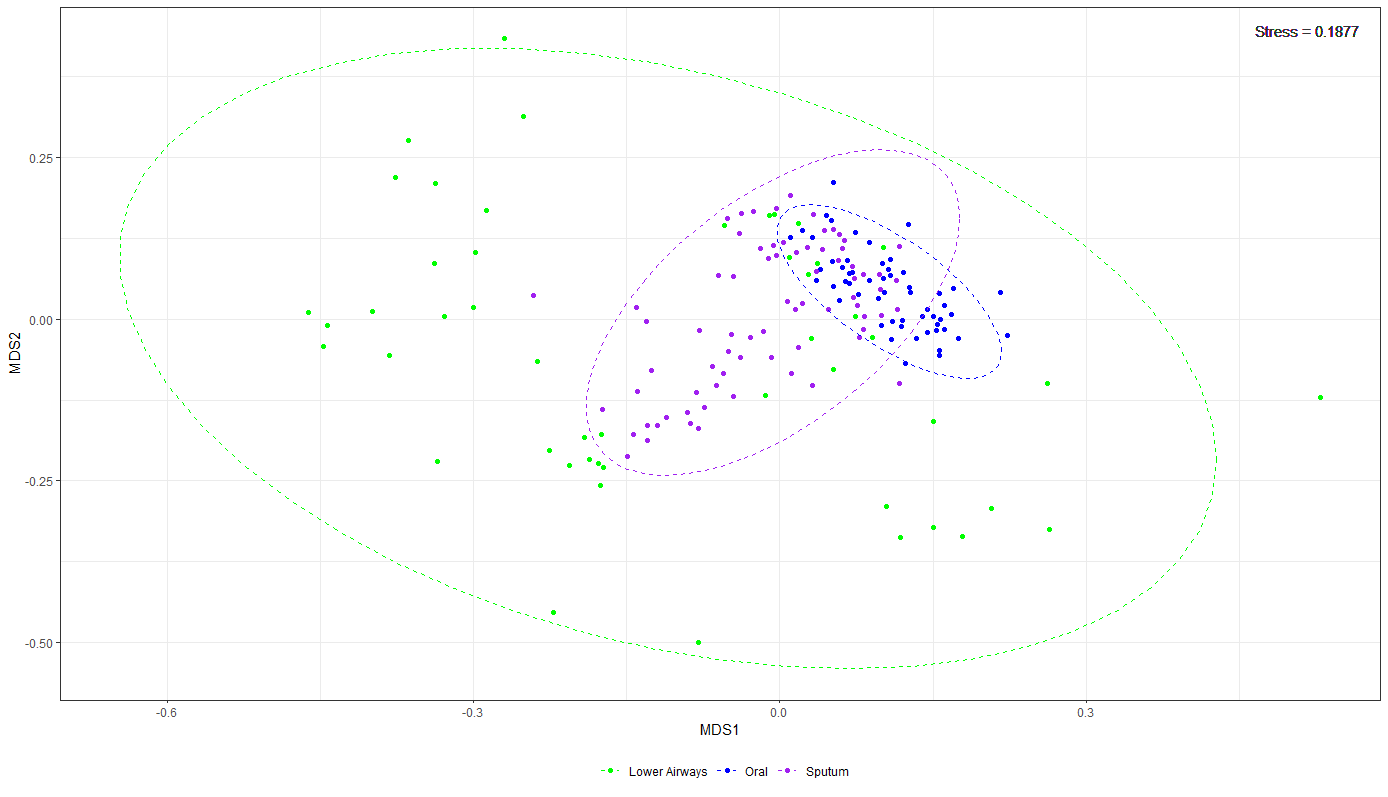

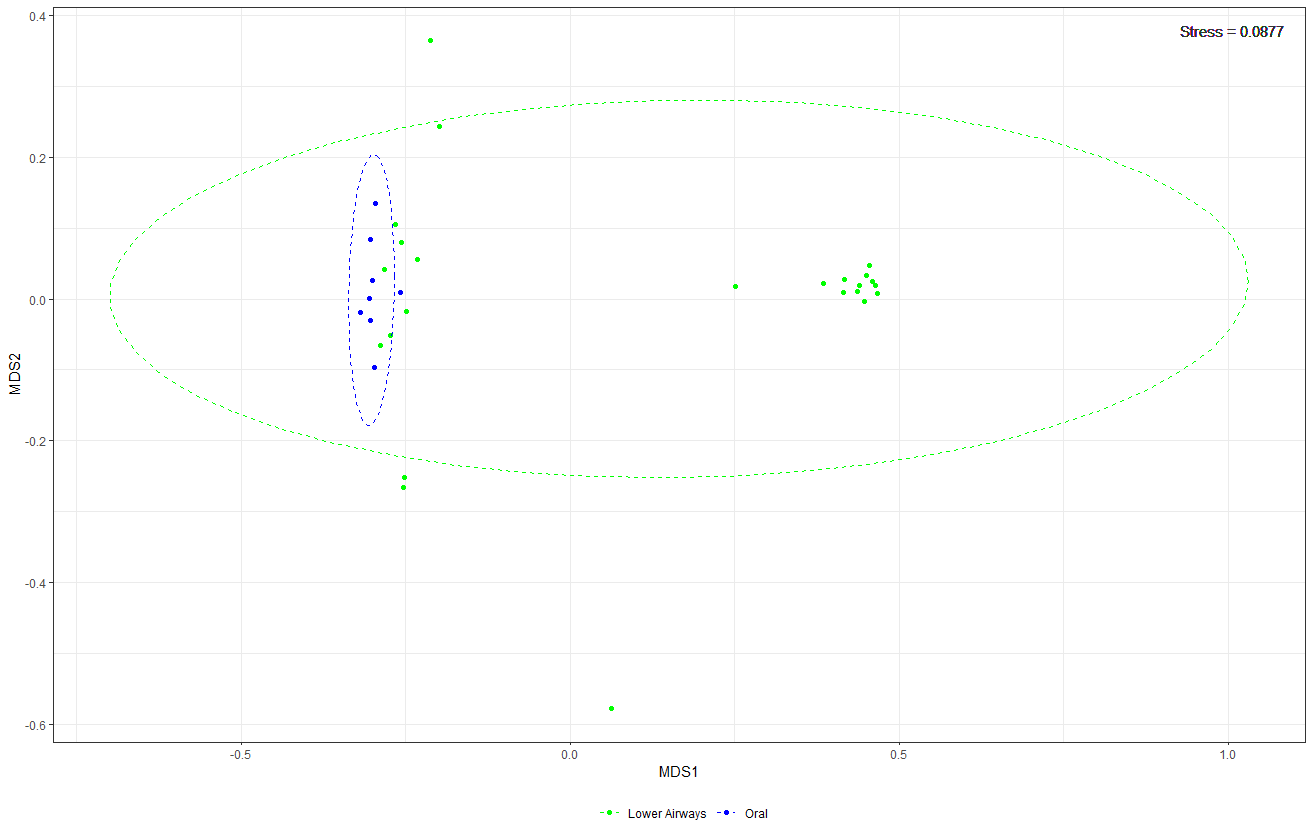


**Figure E4: nMDS plots showing the level of separation of anatomical grouping when only samples from the (top) disease control (n=32), and (bottom) cystic fibrosis (n=180) diagnostic groups are considered.**


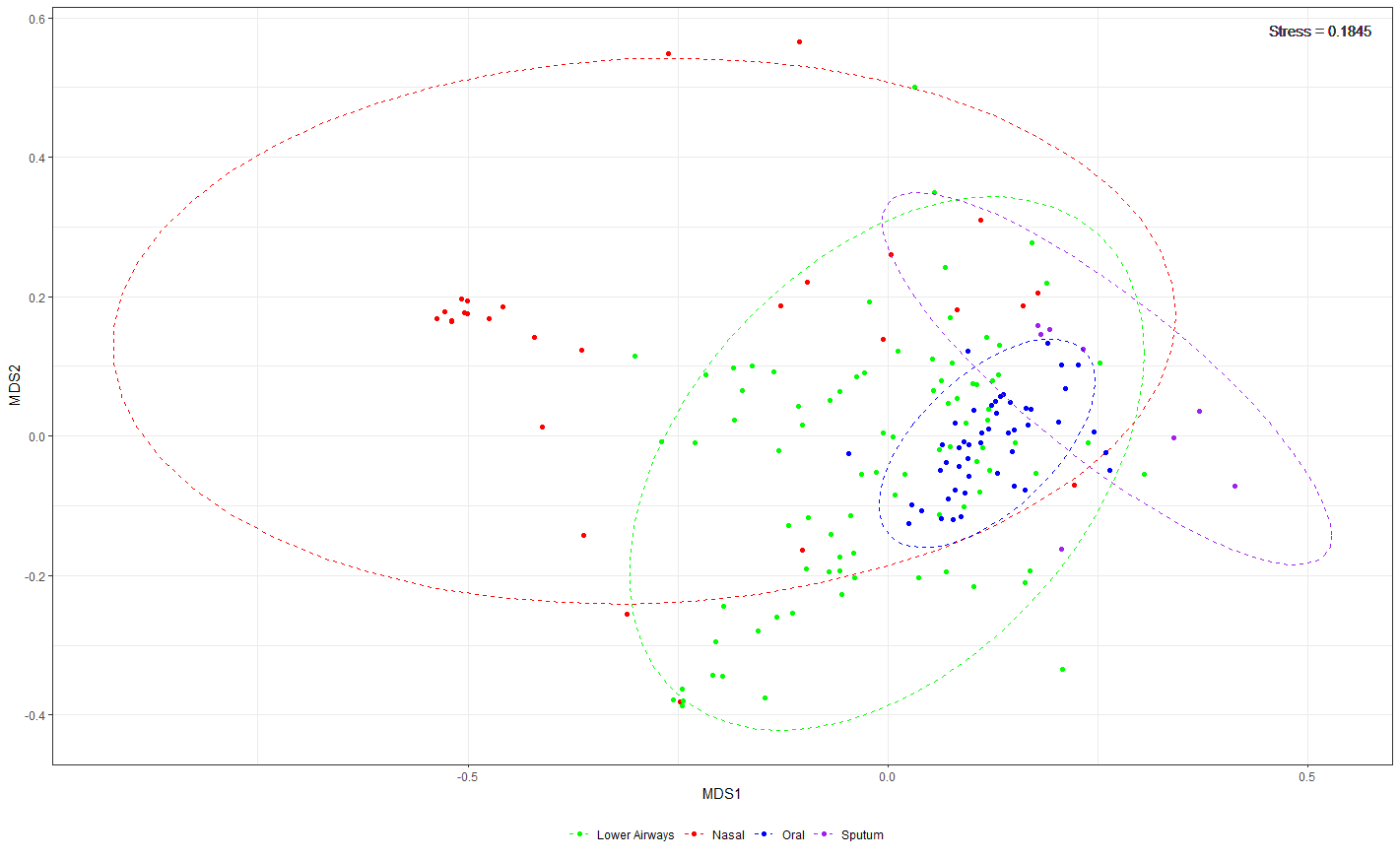

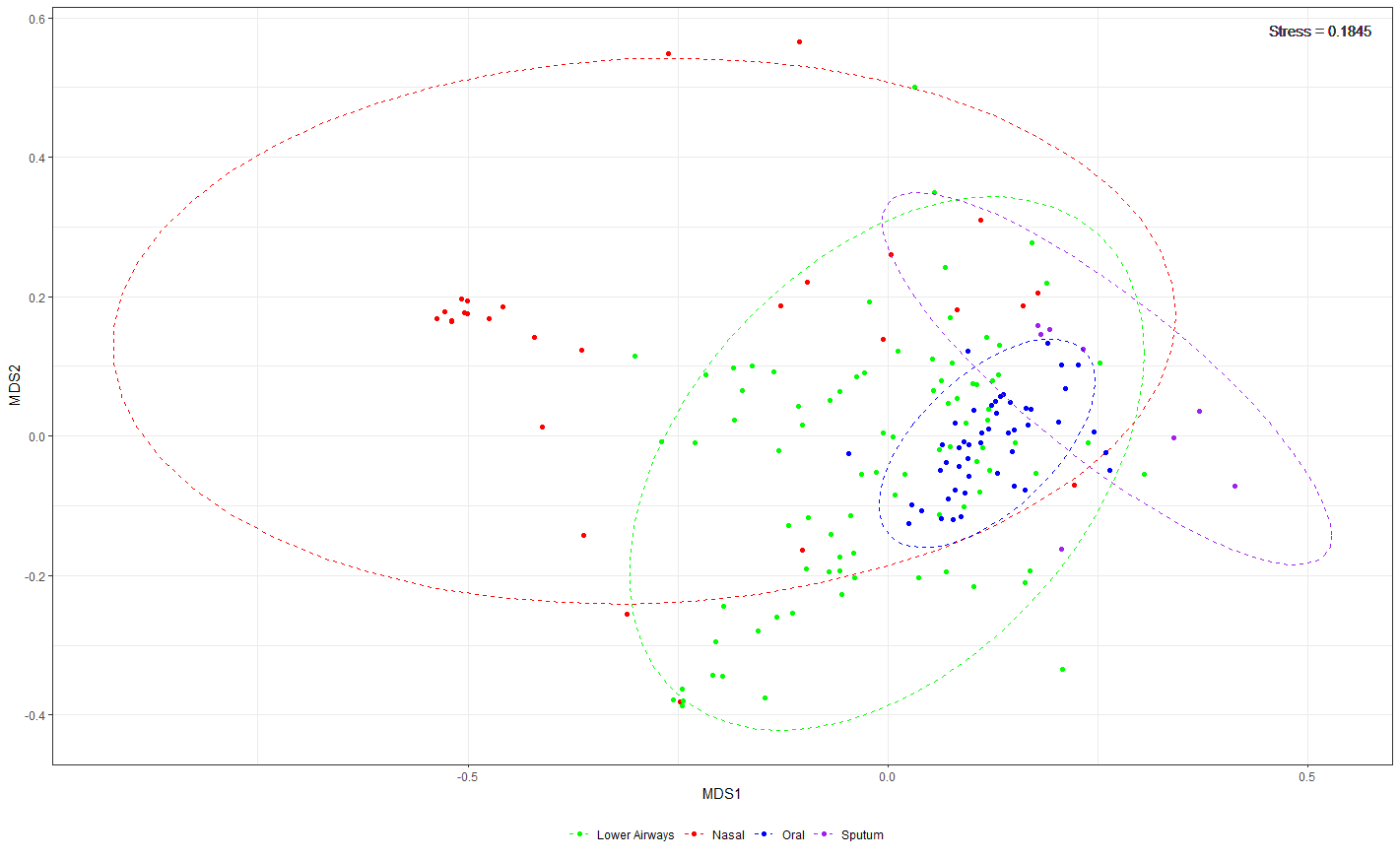

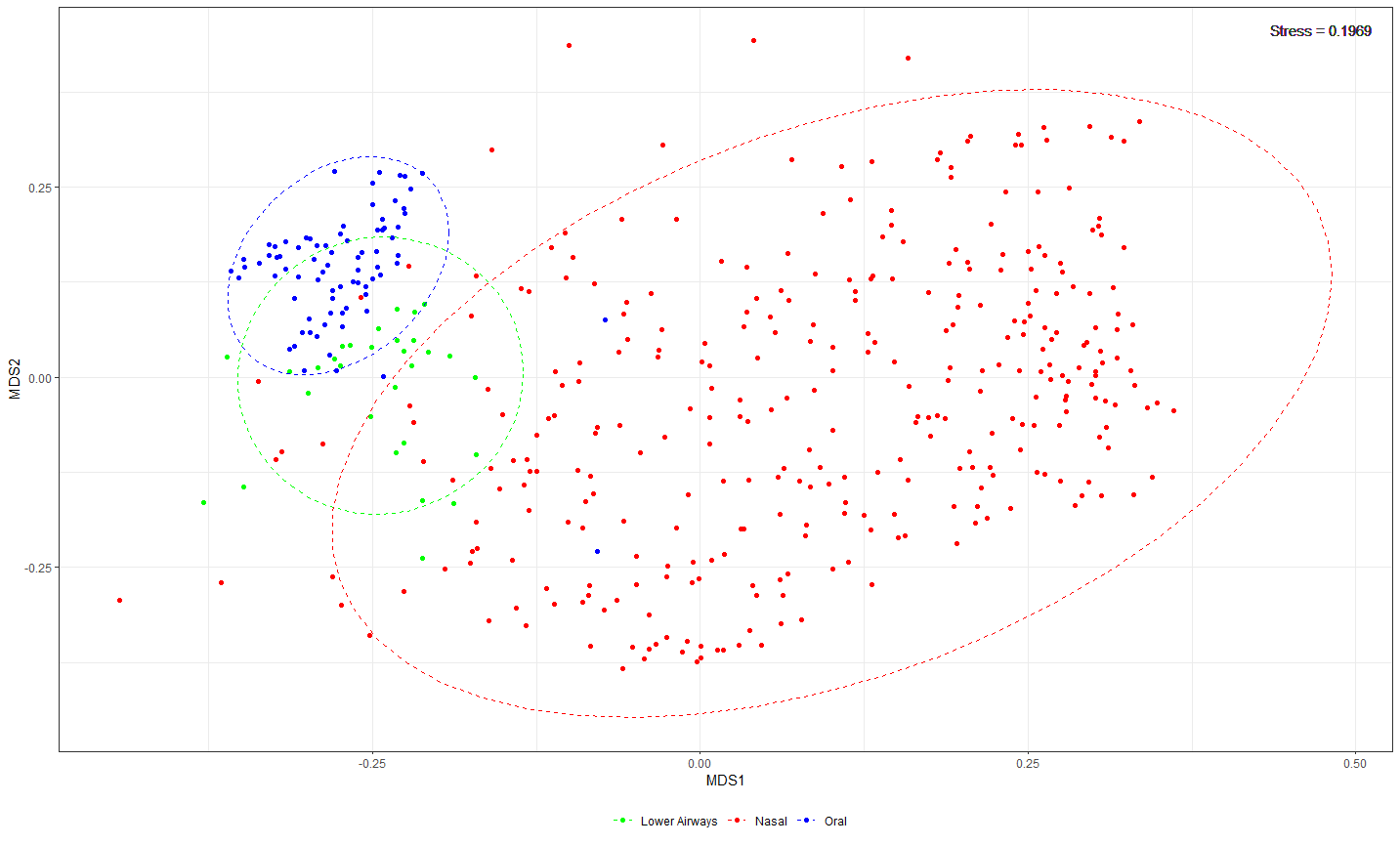


**Figure E5: nMDS plots showing the level of separation of anatomical grouping when only samples from the (top) healthy (n=438) and (bottom) suppurative diseases (n=171) diagnostic groups are considered.**


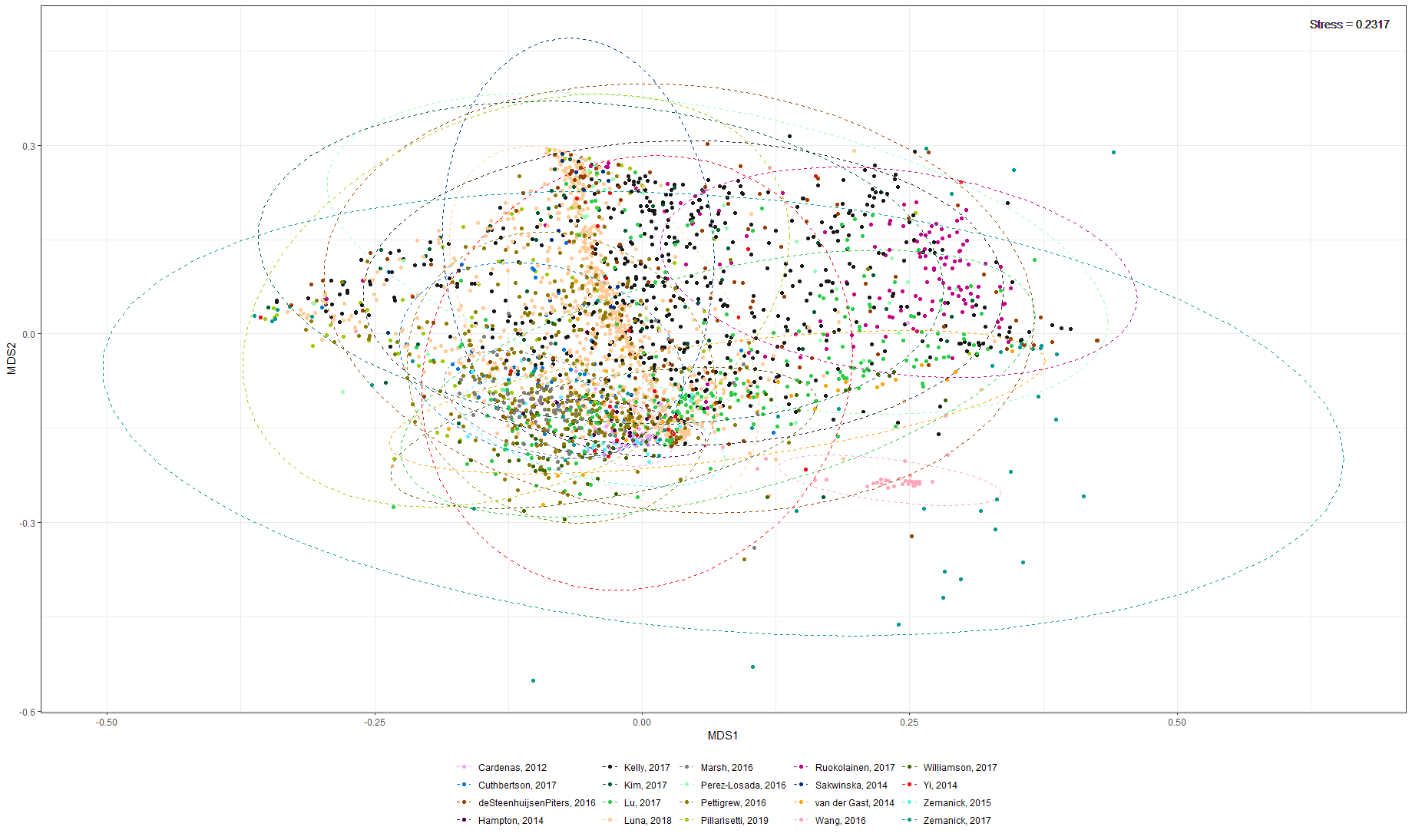

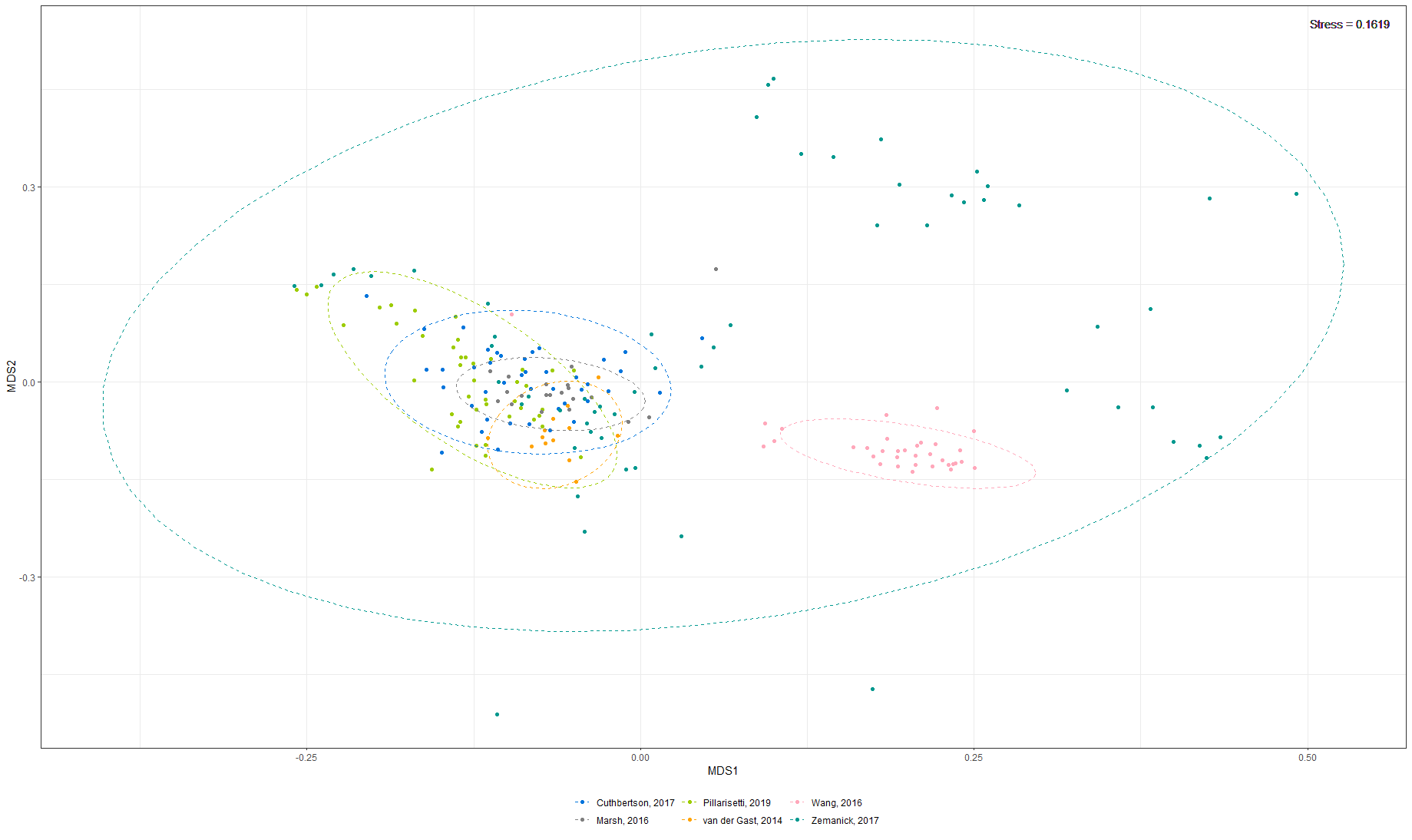

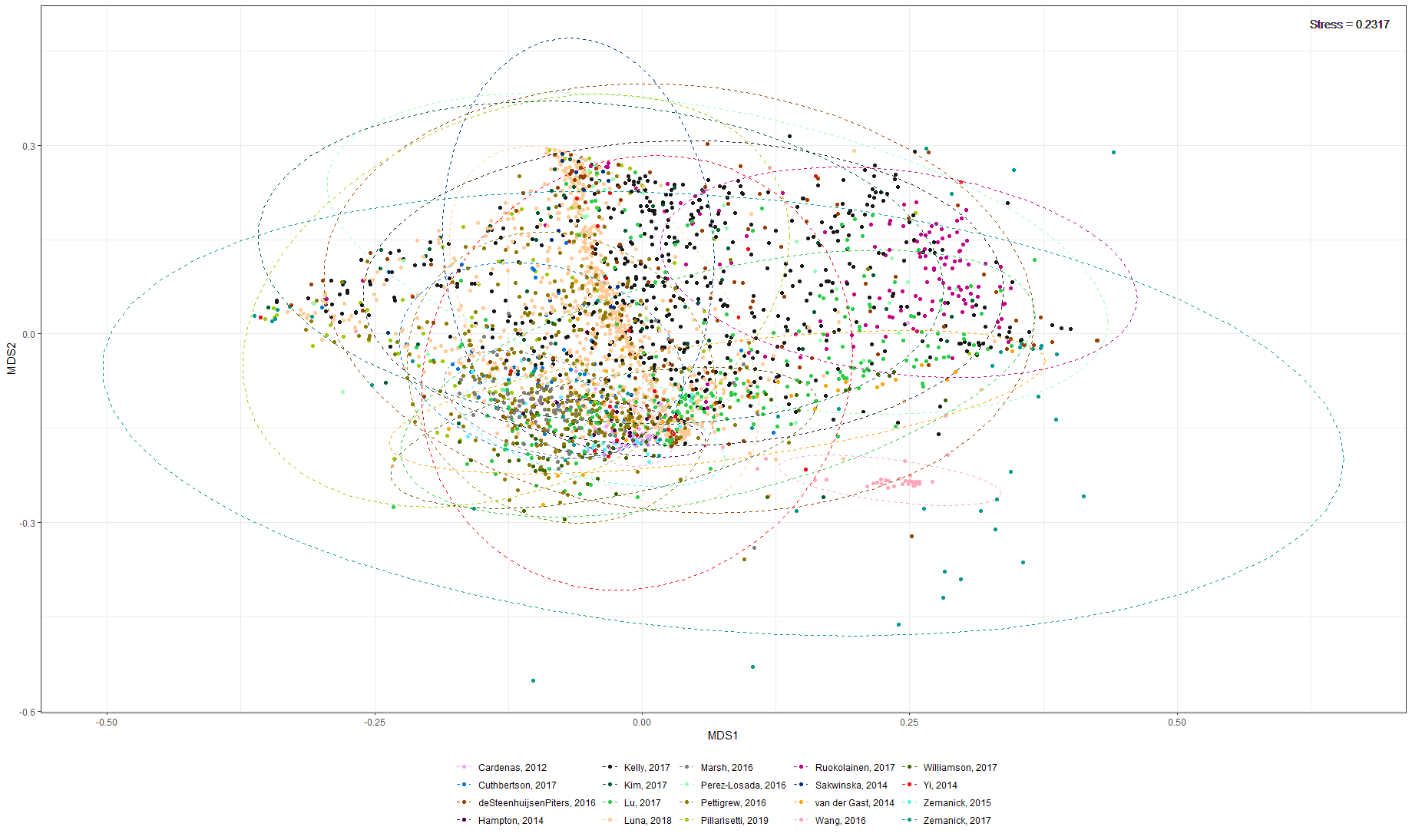


Wang *et al.* cluster

Wang *et al.* cluster

**Figure E6: 16S rRNA gene-based nMDS plot (based on Bray-Curtis dissimilarity) illustrating distinct clustering of the Wang *et al.* 2016 data, in the entire dataset (top) and lower airways dataset only (bottom).** While the reason for this distinct clustering is unclear, as the Wang data are the only included example of lower airway acute infection samples, this warranted caution and its inclusion in the sensitivity analysis.

**Figure E7: Bacterial beta-diversity (i.e. diversity between samples) based on pairwise Bray-Curtis dissimilarity distance of (A) broad diagnostic groupings and (B) specific diagnostic groupings when compared to other groupings which contribute to disease or control groups.**

AI: acute infections, AS: asthma, CF: cystic fibrosis, DC: disease control, HE: healthy, SU: suppurative diseases, WH: wheezing illness

Statistical significance for these comparisons was performed using ANOVA analysis with Tukey’s Honestly Significant Difference test and are presented in Supplementary File B (Tables E18-E24).


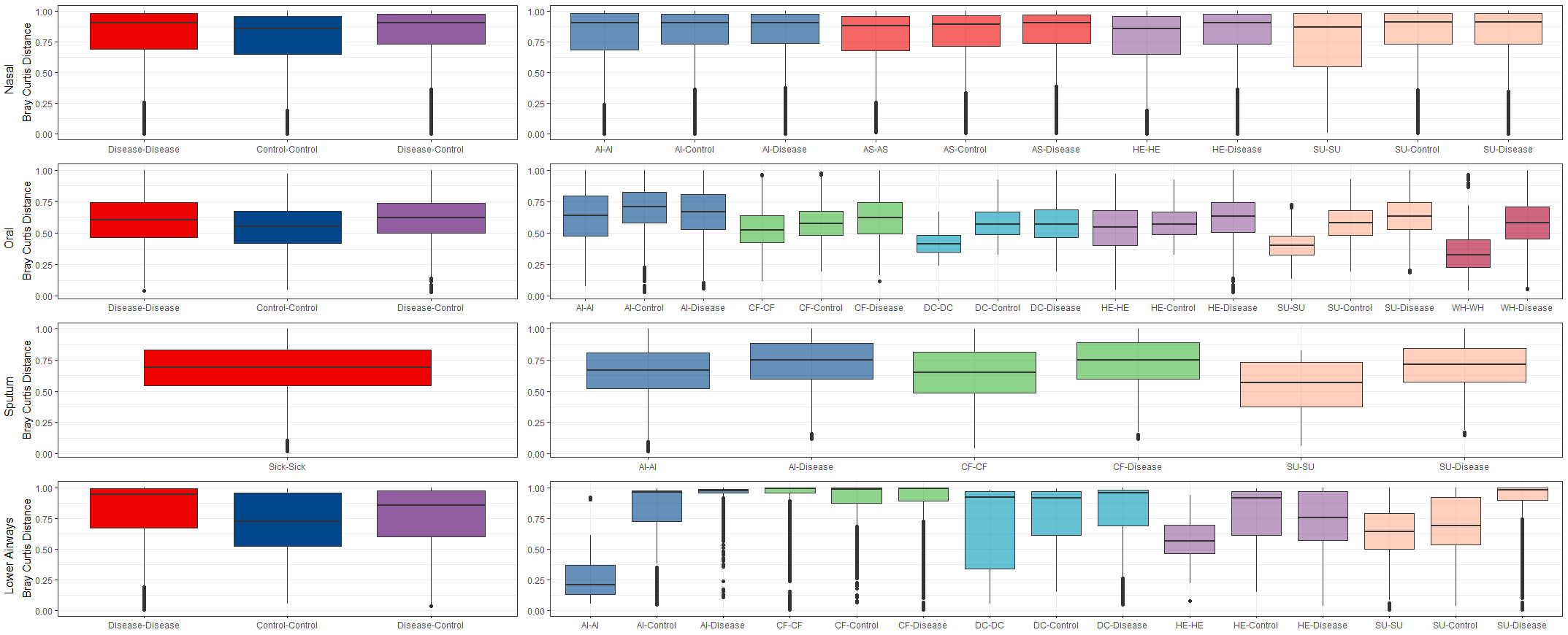


**A**

**B**


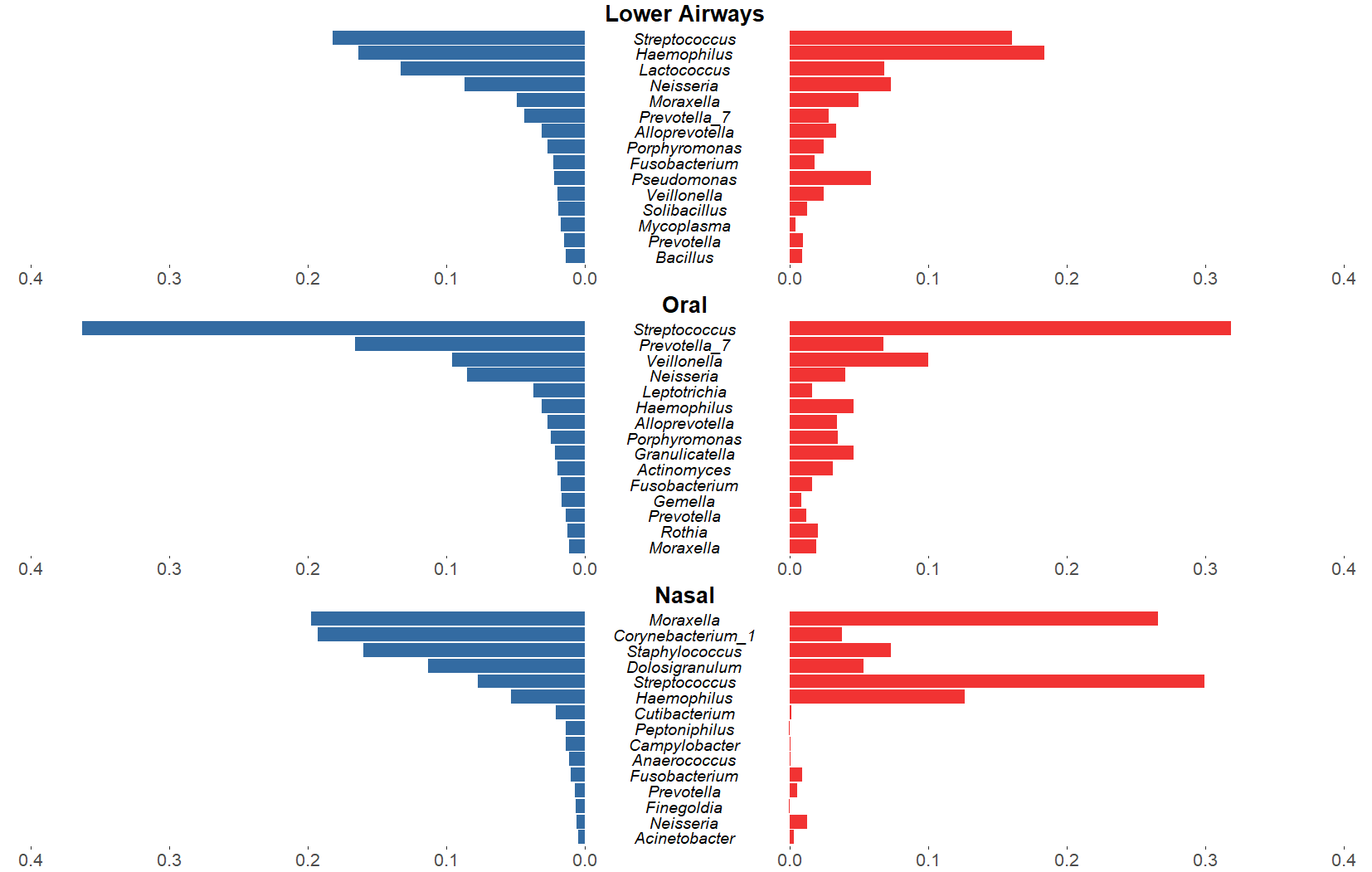

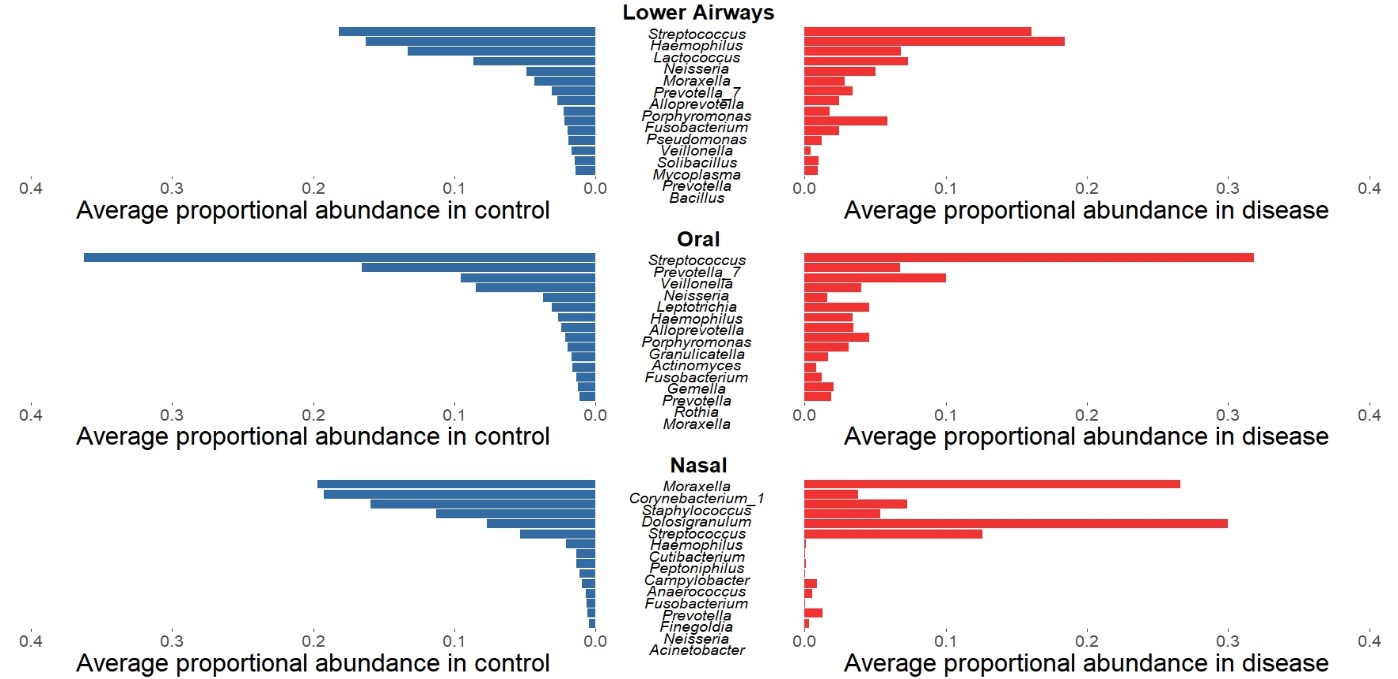

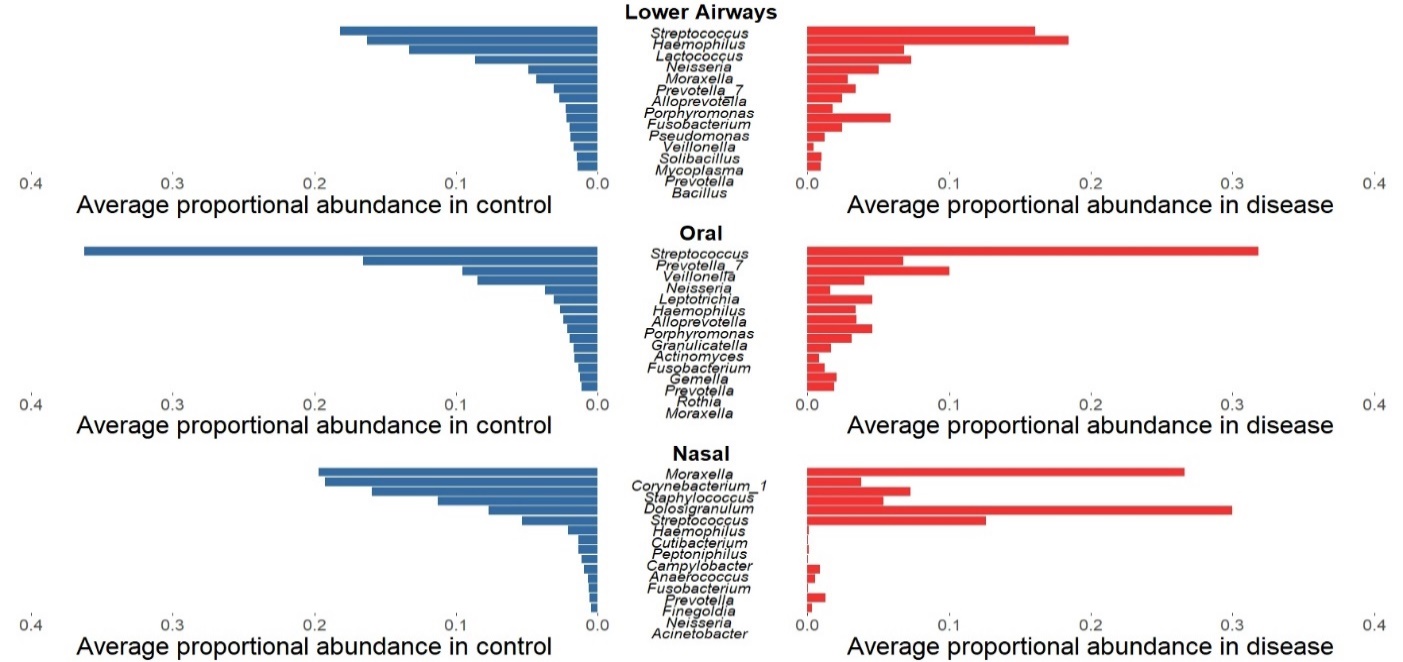


**Figure E8 Rank-abundance plots for rarefied (top) and GMPR (bottom)-normalized datasets.**


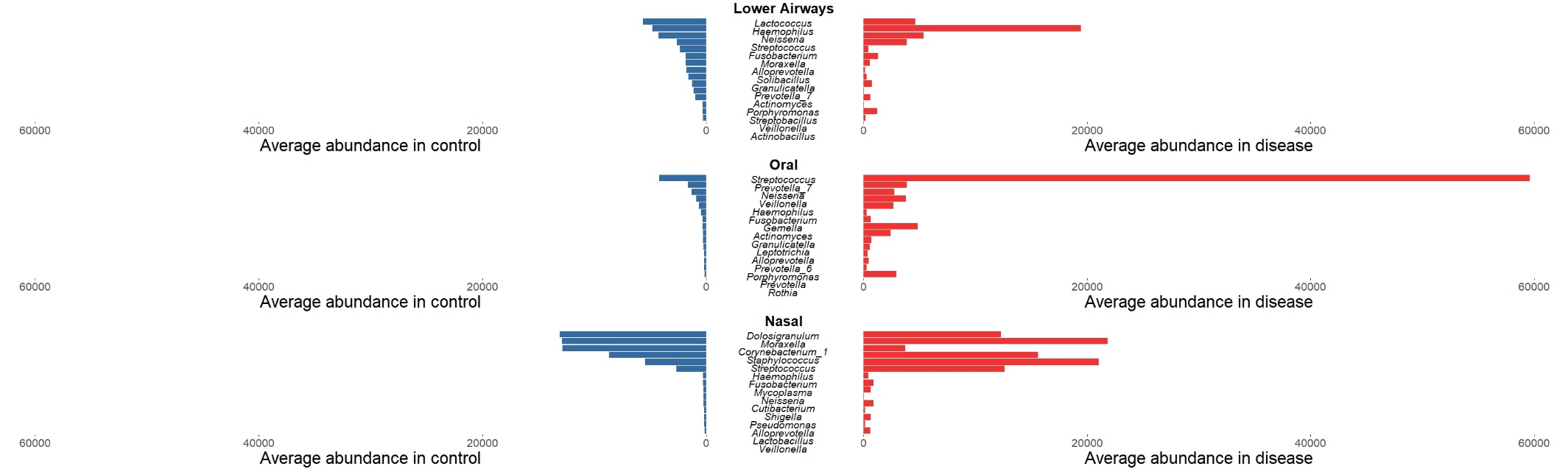

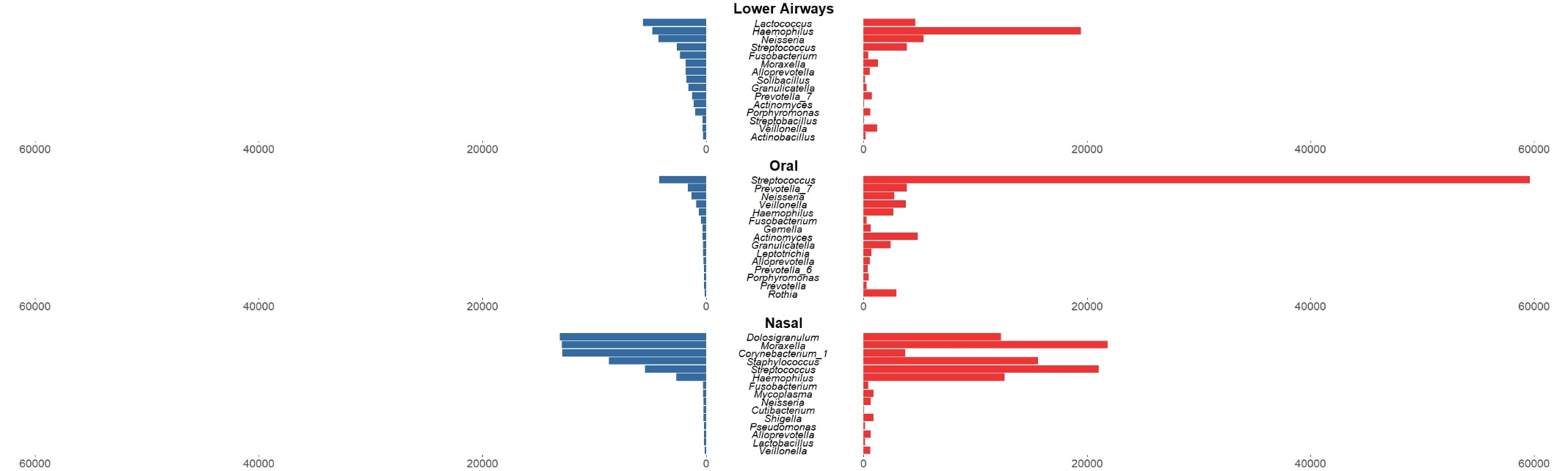

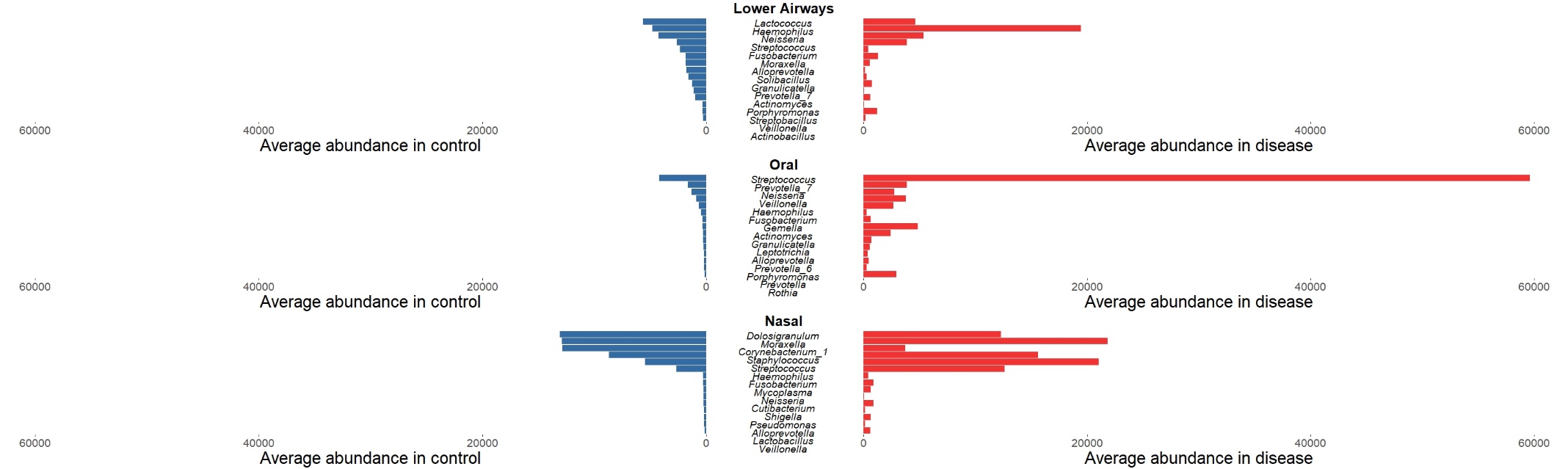


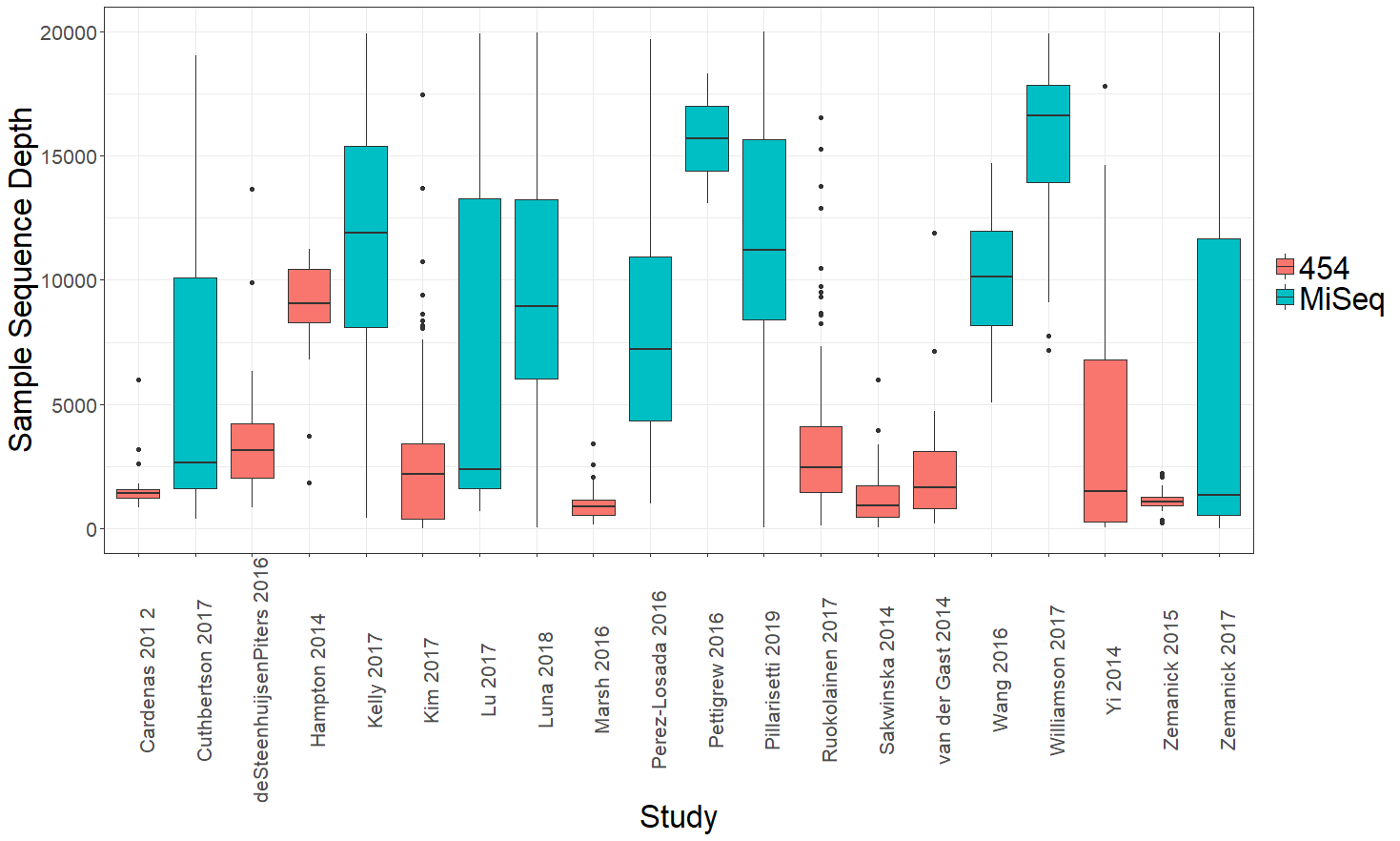


**Figure E9: 16S rRNA gene sequencing depths for each study included in the final dataset, prior to data normalisation.** Red boxplots indicate data derived by 454 pyrosequencing, blue plots indicate data from Illumina MiSeq. To enhance visual clarity the vertical axis is truncated at 20,000 sequences.


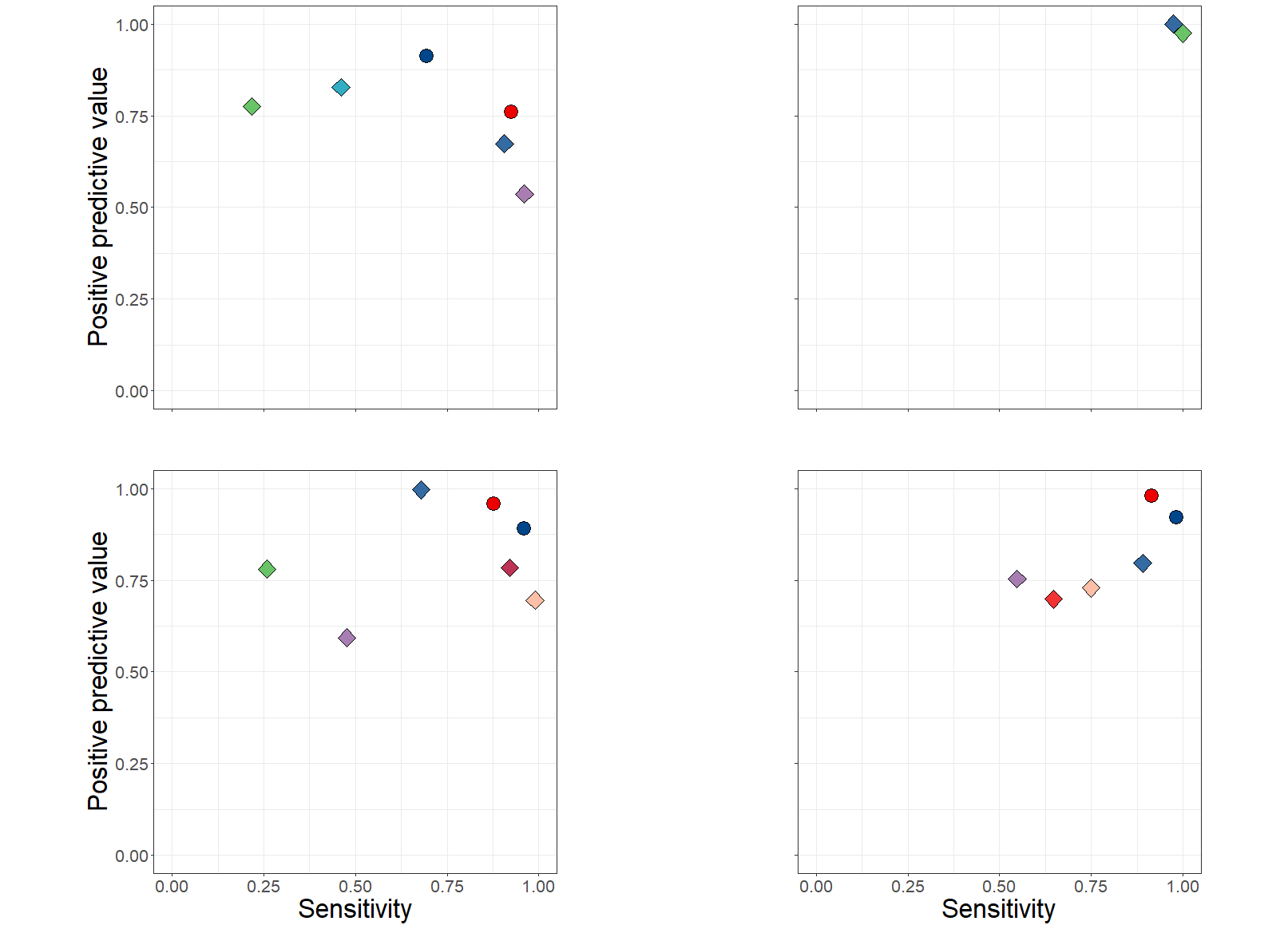

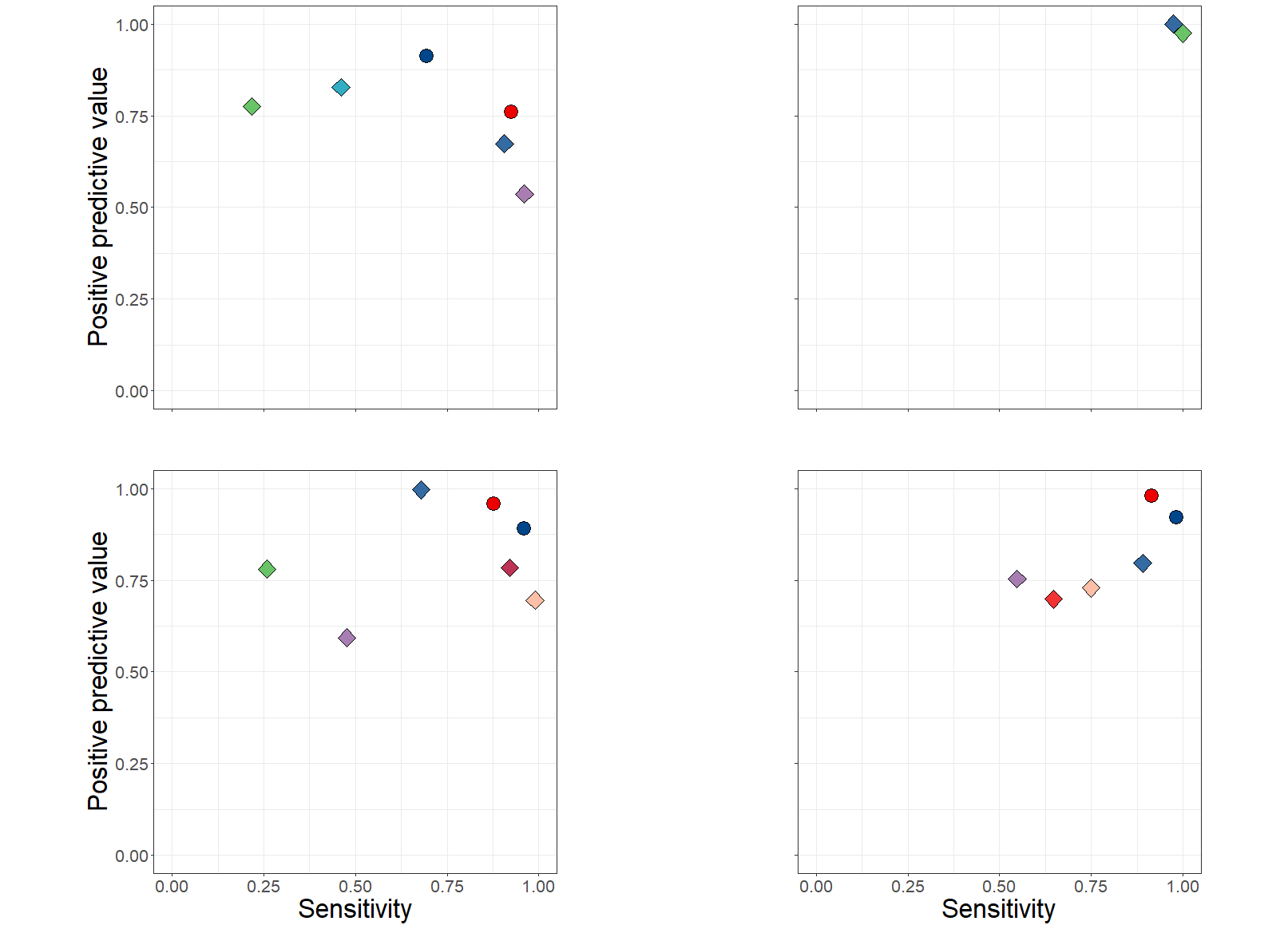

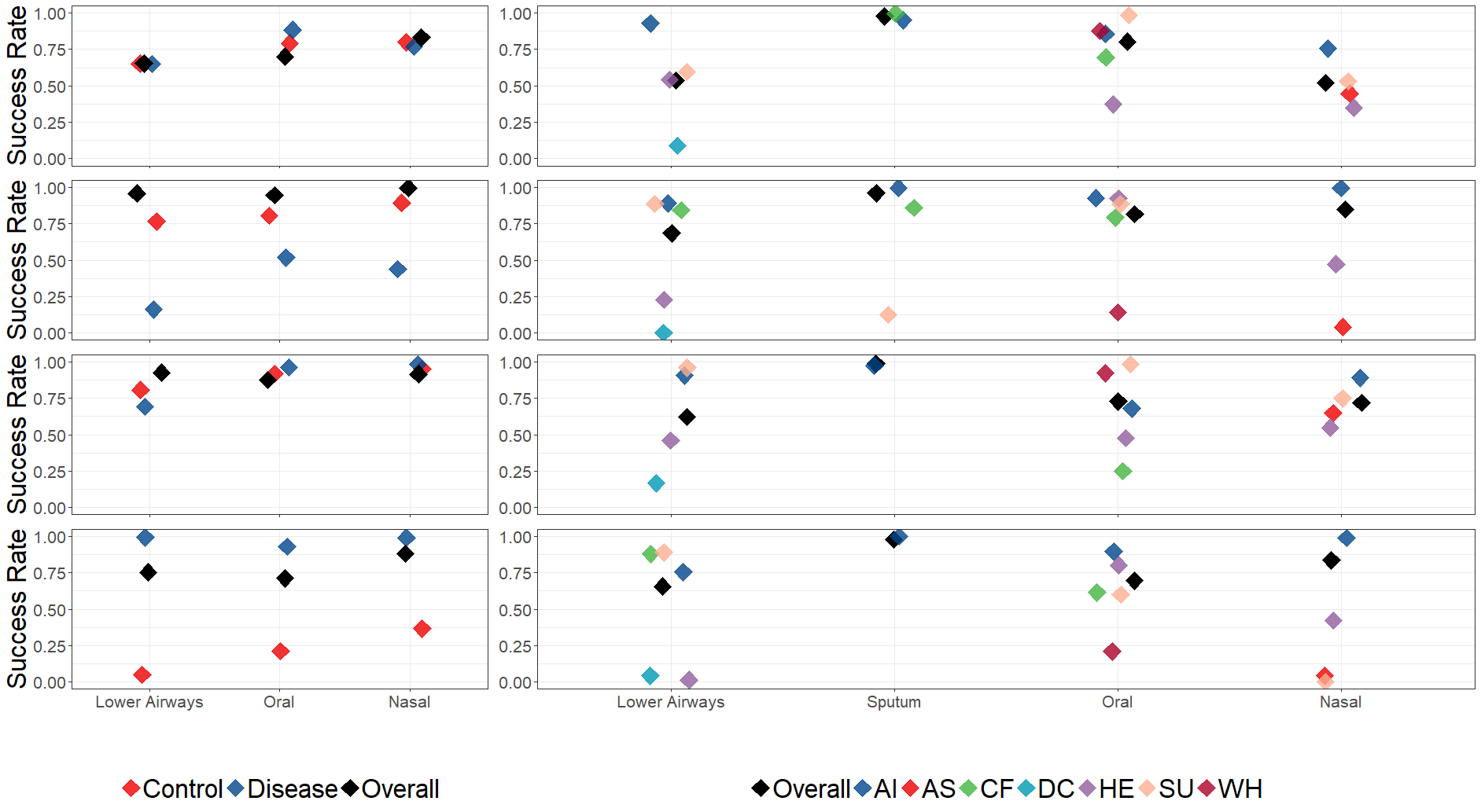


Disease


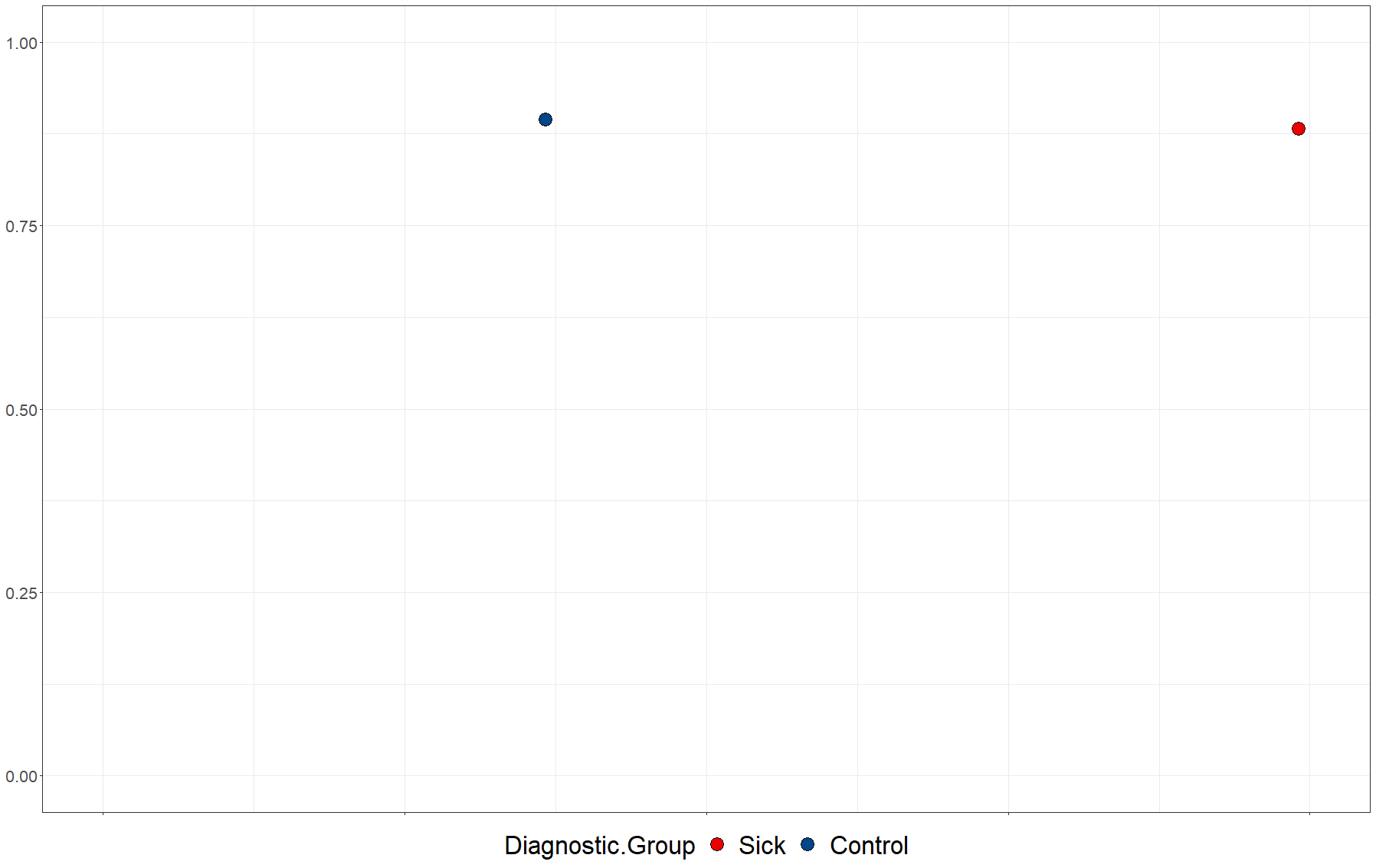


Control


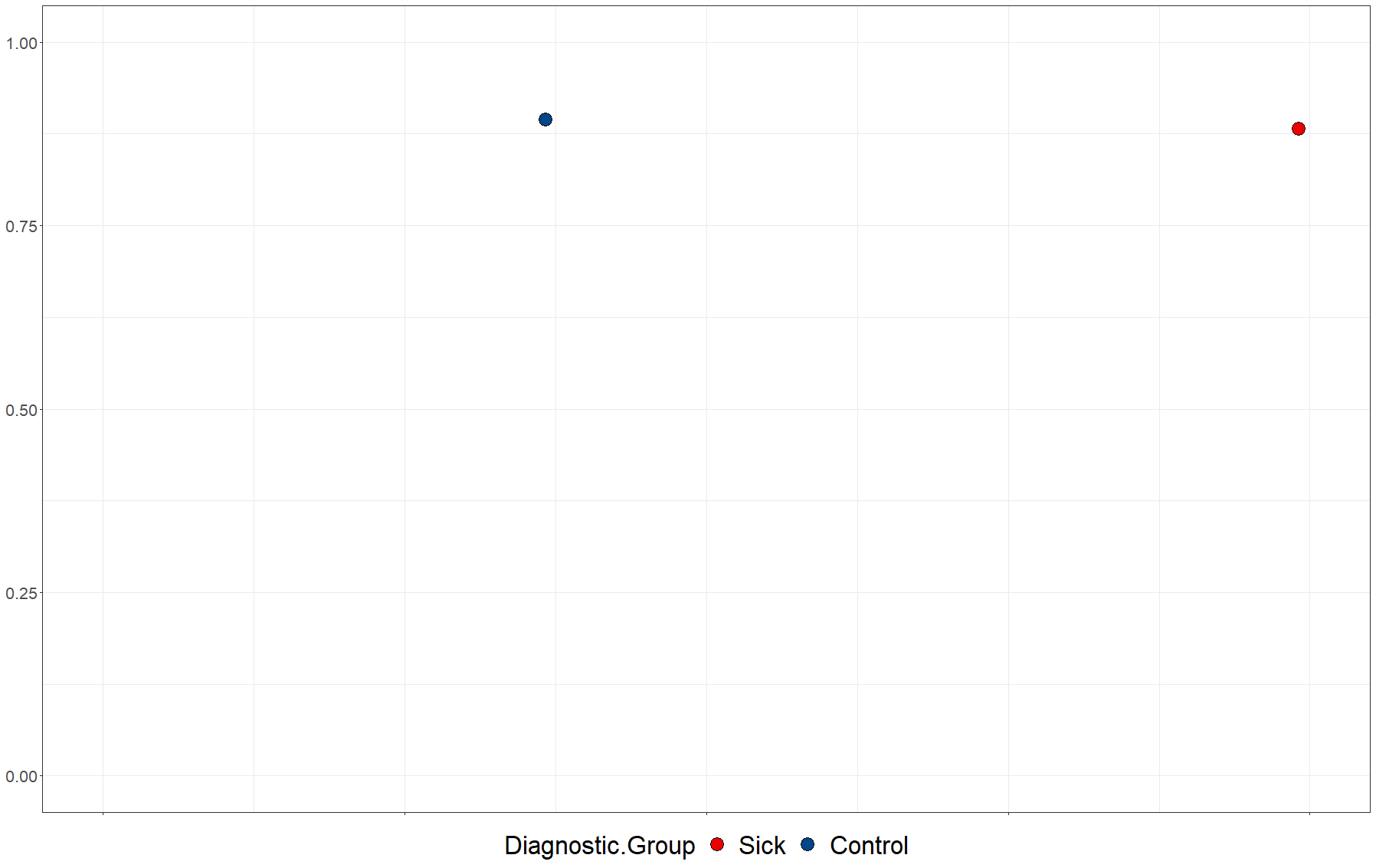


**Nasal**

**Oral**

**Sputum**

**Lower airway**

**Figure E10: Average precision and recall rates of sample assignments to both broad disease level (circles) and specific diagnostic groupings (diamonds), through use of random forest machine learning.** Data are displayed according to anatomical category: nasal, oral, sputum and lower airways. Assignments were made based on GMPR data in which the numbers of samples for each diagnostic grouping were made equal. AI: acute infections, AS: asthma, CF: cystic fibrosis, DC: disease control, HE: healthy, SU: suppurative diseases, WH: wheezing illness

**Bioinformatics Pipeline:**

## B.1 USEARCH and Mothur pipeline example for meta-analysis studies

This pipeline was used to process sequence data included in the meta-analysis and was initially developed by David Waite and used for previous meta-analyses (Waite & Taylor, 2014; Wagner Mackenzie *et al.*, 2017).

#Example Pipeline for standardised pipeline for meta-analysis studies

##Quality Filter Loop

for i in `ls *.fastq | cut -f1-3 -d '_' | sort | uniq`;

do

usearch10.0.240_i86linux64 -fastq_mergepairs ${i}_R1_001.fastq -reverse ${i}_R2_001.fastq -fastqout ../2_Extracted/${i}.fastq

usearch10.0.240_i86linux64 -fastq_filter ../2_Extracted/${i}.fastq -fastq_maxee 1 -fastaout ../2_Extracted/${i}.fasta

done

#Move Fasta Files to a seperate Directory and list

ls *.fasta>fastalist.txt

#Make into count table

mothur

merge.files(input=*FASTA.FILES* output= *COMBINED.FASTA*)

make.group(fasta= *FASTA.FILES*, groups= *FASTA.FILES*, output= *FASTA.GROUPS*)

unique.seqs(fasta= *COMBINED.FASTA*)

count.seqs(name= *COMBINED.NAMES,* group= *FASTA.GROUPS*)

#This data is now ready for the alignment step

# Alignment and filtering

mothur

align.seqs(fasta= *COMBINED.UNIQUE.FASTA*, reference=silva.seed_v132.align, processors=10, flip=T)

screen.seqs(fasta= *COMBINED.UNIQUE.ALIGN*, count= *COMBINED.COUNT_TABLE*, minlength=100, maxhomop=8, processors=10)

summary.seqs(fasta=current, count=current)

#Set sequence ends

screen.seqs(fasta=current, count=current, start=6388, end=13875, maxambig=0)

filter.seqs(fasta=current, vertical=T, trump=.)

summary.seqs(fasta=current, count=current)

# Chimera checking

chimera.uchime(fasta=current, count=current)

remove.seqs(fasta=current, count=current, accnos= *COMBINED.UNIQUE.GOOD.GOOD.FILTER.DENOVO.UCHIME.ACCNOS*.)

# Classification

filter.seqs(fasta=silva.nr_v132.align, hard=8_ *COMBINED.FILTER*)

classify.seqs(fasta= *COMBINED.UNIQUE.GOOD.GOOD.FILTER.PICK.FASTA*, count=current, template=silva.nr_v132.filter.fasta, taxonomy=silva.nr_v132.tax, cutoff=80)

remove.lineage(fasta=current, count=current, taxonomy=current, taxon=Mitochondria-Chloroplast-Archaea-unknown-Bacteria;unknown)

quit

# Check taxonomy, rename final files

cut -f2 = *COMBINED.UNIQUE.GOOD.GOOD.FILTER.PICK.NR_V132.WANG.PICK.TAXONOMY* | cut -f2 -d ';' | cut -f1 -d '(' | sort | uniq -c

mv *COMBINED.UNIQUE.GOOD.GOOD.FILTER.PICK.PICK.FASTA*../8_ *COMBINED.FINAL.FASTA*

mv *COMBINED.GOOD.GOOD.PICK.PICK.COUNT_TABLE*../ *COMBINED.FINAL.COUNT_TABLE*

mv *COMBINED.UNIQUE.GOOD.GOOD.FILTER.PICK.NR_V132.WANG.PICK.TAXONOMY*../ *COMBINED.FINAL.TAXONOMY*

mv *COMBINED.FILTER*../ *COMBINED.FILTER*

# Clean out the database files

rm ~/MothurUpdated/mothur/silva.filter

rm ~/MothurUpdated/mothur/silva.nr_v132.tree.sum

rm ~/MothurUpdated/mothur/silva.nr_v132.filter.8mer

rm ~/MothurUpdated/mothur/silva.nr_v132.tree.train

rm ~/MothurUpdated/mothur/silva.nr_v132.filter.fasta

rm ~/MothurUpdated/mothur/silva.nr_v132.silva.nr_v132.filter.8mer.numNonZero

rm ~/MothurUpdated/mothur/silva.nr_v132.silva.nr_v132.filter.8mer.prob

## B.2 Meta-analysis machine learning code

This code was run in python using the anaconda platform, developed by David Waite and David Broderick.

#Set Directory

cd Desktop

cd MachineLearning

cd CombSensDat

#Start Python

python

# Model imports

from sklearn import svm

from sklearn.ensemble import RandomForestClassifier

from sklearn.neural_network import MLPClassifier

# Useful for creating data splits for cross-fold validation

from sklearn.model_selection import StratifiedShuffleSplit

# Needed for saving models

from sklearn.externals import joblib

# matthews_corrcoef is my preferred metric for evaluating models

from sklearn.metrics import matthews_corrcoef

import pandas as pd

otu_df = pd.read_csv('Nasal.SickML.NoLuna.SeqDat.csv', sep=',').set_index('Seq_ID')

otu_metadata = pd.read_csv('Nasal.SickML.NoLuna.MetDat.csv').set_index('Seq_ID').reindex( otu_df.index )

otu_metadata = list( otu_metadata.Sick )

def pull_sample_name(file_name):

return file_name.replace('SickML.NoLuna.SeqDat.csv', '')

def yield_dataframe_splits(_df, _metadata, n_splits, test_size, seed, return_indices=False):

sss_obj = StratifiedShuffleSplit(n_splits=n_splits, test_size=test_size, random_state=seed)

for i, (tr, te) in enumerate( sss_obj.split(_df, _metadata) ):

# Split data, tr indices for training, te indices for testing

training_samples = _df.iloc[ tr, : ]

training_labels = [ _metadata[x] for x in tr ]

testing_samples = _df.iloc[ te, : ]

testing_labels = [ _metadata[x] for x in te ]

if return_indices:

yield i, training_samples, training_labels, testing_samples, testing_labels, te

else:

yield i, training_samples, training_labels, testing_samples, testing_labels

def log_calls(samples, exp_values, obs_values, output_name):

df = pd.DataFrame( { 'Samples': samples, 'Labels': exp_values, 'Predictions': obs_values } )

df.to_csv(output_name, sep='\t', index=False)

import warnings

import numpy as np

def masked_mcc_score(exp_values, obs_values):

with warnings.catch_warnings(record=True) as w:

mcc = matthews_corrcoef(exp_values, obs_values)

return np.NaN if len(w) > 0 else mcc

sample_name = pull_sample_name('Nasal.SickML.NoLuna.SeqDat.csv')

model_scores = []

for i, tr_df, tr_l, te_df, te_l, te in yield_dataframe_splits(otu_df, otu_metadata, 10, 0.5, 12345, return_indices=True):

# Train the model

machine_model = machine_model = RandomForestClassifier(n_estimators=1000)

_ = machine_model.fit(tr_df, tr_l)

# Evaluate the model

predictions = machine_model.predict(te_df)

model_scores.append( { 'Permutation': i, 'MCC': masked_mcc_score(te_l, predictions) } )

# Log model and specific calls

joblib.dump(machine_model, '{}.{}.pkl'.format(sample_name, i) )

log_calls(otu_df.iloc[te,:].index, te_l, predictions, '{}.{}.txt'.format(sample_name, i))

# Once the loop completes, write out all of the MCC score for the various models

pd.DataFrame(model_scores).to_csv('{}.mcc.txt'.format(sample_name), sep='\t', index=False)

**References**

Cardenas PA, Cooper PJ, Cox MJ, Chico M, Arias C, Moffatt MF, Cookson WO. Upper airways microbiota in antibiotic-naive wheezing and healthy infants from the tropics of rural Ecuador. *PLoS One* 2012; **7**(10): e46803.

Chen L, Reeve J, Zhang L, Huang S, Wang X, Chen J. GMPR: A robust normalisation method for zero-inflated count data with application to microbiome sequencing data. *PeerJ* 2018; **6**: e4600.

Cuthbertson L, Craven V, Bingle L, Cookson, William O. C. M., Everard ML, Moffatt MF. The impact of persistent bacterial bronchitis on the pulmonary microbiome of children. *PLoS One* 2017; **12**(12): e0190075.

Deeks JJ, Higgins JP, Altman DG, Cochrane Statistical Methods Group. Analysing data and undertaking meta‐analyses. In: Higgins JPT, Thomas J, Chandler J *et al.*, (eds.) *Cochrane Handbook for Systematic Reviews of Interventions* 2019. London, UK: Cochrane.

de Steenhuijsen Piters, Heinonen S, Hasrat R, et al. Nasopharyngeal microbiota, host transcriptome, and disease severity in children with respiratory syncytial virus infection. *Am J Respir Crit Care Med* 2016; **194**(9): 1104-15.

Edgar RC. Search and clustering orders of magnitude faster than BLAST. *Bioinformatics* 2010; **26**(19): 2460-1.

Edgar RC, Haas BJ, Clemente JC, Quince C, Knight R. UCHIME improves sensitivity and speed of chimera detection. *Bioinformatics* 2011; **27**(16): 2194-200.

Hampton TH, Green DM, Cutting GR, et al. The microbiome in pediatric cystic fibrosis patients: the role of shared environment suggests a window of intervention. *Microbiome* 2014; **2**(1): 14.

Kelly MS, Surette MG, Smieja M, et al. The nasopharyngeal microbiota of children with respiratory infections in Botswana. *Pediatr Infect Dis J* 2017; **36**(9).

Kim B‐, Lee E, Lee M‐, et al. Different functional genes of upper airway microbiome associated with natural course of childhood asthma. *Allergy* 2017; **73**(3): 644-52.

Langevin S, Pichon M, Smith E, et al. Early nasopharyngeal microbial signature associated with severe influenza in children: a retrospective pilot study. *J Gen Virol* 2017; **98**(10): 2425-37.

Lu Z, Dai W, Liu Y, et al. The alteration of nasopharyngeal and oropharyngeal microbiota in children with MPP and non-MPP. *Genes* 2017; **8**.

Luna PN, Hasegawa K, Ajami NJ, et al. The association between anterior nares and nasopharyngeal microbiota in infants hospitalized for bronchiolitis. *Microbiome* 2018; **6**(1): 2.

Man WH, de Steenhuijsen Piters, Wouter AA, Bogaert D. The microbiota of the respiratory tract: gatekeeper to respiratory health. *Nat Rev Microbiol* 2017; **15**(5): 259.

Marsh RL, Kaestli M, Chang AB, Binks MJ, Pope CE, Hoffman LR, Smith-Vaughan H. The microbiota in bronchoalveolar lavage from young children with chronic lung disease includes taxa present in both the oropharynx and nasopharynx. *Microbiome* 2016; **4**(1): 37.

Pedregosa F, Varoquaux G, Gramfort A, et al. Scikit-learn: Machine learning in Python. *J Mach Learn Res* 2011; **12**: 2825-30.

Perez-Losada M, Crandall KA, Freishtat RJ. Two sampling methods yield distinct microbial signatures in the nasopharynges of asthmatic children. *Microbiome* 2016; **4**(1): 25.

Pettigrew MM, Gent JF, Kong Y, et al. Association of sputum microbiota profiles with severity of community-acquired pneumonia in children. *BMC Infect Dis* 2016; **16**(1): 317.

Pillarisetti N, Broderick D, Ainsworth A, et al. The airway microbiota in children newly diagnosed with bronchiectasis largely retains its diversity. *Eur Respir J* 2019; **54**(2): 1900704.

Quast C, Pruesse E, Yilmaz P, et al. The SILVA ribosomal RNA gene database project: improved data processing and web-based tools. *Nucleic Acids Res* 2012; **41**(D1): D590-6.

R Core Team. R: A Language and Environment for Statistical Computing. *Vienna, Austria* 2019.

Ronchetti K, Tame J, Paisey C, et al. The CF-Sputum Induction Trial (CF-SpIT) to assess lower airway bacterial sampling in young children with cystic fibrosis: a prospective internally controlled interventional trial. *Lancet Respir Med* 2018; **6**(6): 461-71.

Ruokolainen L, Paalanen L, Karkman A, et al. Significant disparities in allergy prevalence and microbiota between the young people in Finnish and Russian Karelia. *Clin Exp Allergy* 2017; **47**(5): 665-74.

Sakwinska O, Bastic Schmid V, Berger B, et al. Nasopharyngeal microbiota in healthy children and pneumonia patients. *J Clin Microbiol* 2014; **52**(5): 1590-4.

Schloss PD, Westcott SL, Ryabin T, et al. Introducing mothur: open-source, platform-independent, community-supported software for describing and comparing microbial communities. *Appl Environ Microbiol* 2009; **75**(23): 7537-41.

Schwartz AG, Yang P, Swanson GM. Familial risk of lung cancer among nonsmokers and their relatives. *Am J Epidemiol* 1996; **144**(6): 554-62.

Segata N, Izard J, Waldron L, Gevers D, Miropolsky L, Garrett WS, Huttenhower C. Metagenomic biomarker discovery and explanation. *Genome Biol* 2011; **12**(6): R60.

Sokolova M, Japkowicz N, Szpakowicz S. Beyond accuracy, F-score and ROC: a family of discriminant measures for performance evaluation. Australasian joint conference on artificial intelligence; Springer; 2006.

van der Gast C, Cuthbertson L, Rogers GB, et al. Three clinically distinct chronic pediatric airway infections share a common core microbiota. *Annals ATS* 2014; **11**(7): 1039-48.

Wang H, Dai W, Qiu C, et al. *Mycoplasma pneumoniae* and *Streptococcus pneumoniae* caused different microbial structure and correlation network in lung microbiota. *Journal of Thoracic Disease* 2016; **8**(6): 1316-22.

Wickham H. ggplot2: elegant graphics for data analysis. Springer; 2016.

Williamson KM, Wagner BD, Robertson CE, Johnson EJ, Zemanick ET, Harris JK. Impact of enzymatic digestion on bacterial community composition in CF airway samples. *PeerJ* 2017; **5**: e3362

Yi H, Yong D, Lee K, Cho Y, Chun J. Profiling bacterial community in upper respiratory tracts. *BMC Infect Dis* 2014; **14**(1): 583.

Zemanick ET, Wagner BD, Robertson CE, et al. Assessment of airway microbiota and inflammation in cystic fibrosis using multiple sampling methods. *Annals ATS* 2015; **12**(2): 221-9.

Zemanick ET, Wagner BD, Robertson CE, et al. Airway microbiota across age and disease spectrum in cystic fibrosis. *Eur Respir J* 2017; **50**(5).
